# The impact of p53 on aristolochic acid I-induced nephrotoxicity and DNA damage in vivo and in vitro

**DOI:** 10.1007/s00204-019-02578-4

**Published:** 2019-10-10

**Authors:** Mateja Sborchia, Eric G. De Prez, Marie-Hélène Antoine, Lucie Bienfait, Radek Indra, Gabriel Valbuena, David H. Phillips, Joëlle L. Nortier, Marie Stiborová, Hector C. Keun, Volker M. Arlt

**Affiliations:** 1 Department of Analytical, Environmental and Forensic Sciences, MRC-PHE Centre for Environment and Health, King’s College London, London, SE1 9NH UK; 2 Laboratory of Experimental Nephrology, Department of Experimental Biochemistry, Faculty of Medicine, Université Libre de Bruxelles, 1070 Brussels, Belgium; 3 Department of Pathology, Erasme University Hospital, 1070 Brussels, Belgium; 4 Department of Biochemistry, Faculty of Science, Charles University Prague, 128 40, Prague, Czech Republic; 5 Department of Surgery and Cancer, Faculty of Medicine, Imperial College London, London, W12 0NN UK

**Keywords:** Aristolochic acid I, Tumour suppressor p53, Mouse models, Carcinogen metabolism, DNA adducts, Mouse embryonic fibroblasts

## Abstract

Exposure to aristolochic acid (AA) is associated with human nephropathy and urothelial cancer. The tumour suppressor *TP53* is a critical gene in carcinogenesis and frequently mutated in AA-induced urothelial tumours. We investigated the impact of p53 on AAI-induced nephrotoxicity and DNA damage in vivo by treating *Trp53*(+*/*+), *Trp53*(+*/*−) and *Trp53*(−*/*−) mice with 3.5 mg/kg body weight (bw) AAI daily for 2 or 6 days. Renal histopathology showed a gradient of intensity in proximal tubular injury from *Trp53*(+*/*+) to *Trp53*(−*/*−) mice, especially after 6 days. The observed renal injury was supported by nuclear magnetic resonance (NMR)-based metabonomic measurements, where a consistent *Trp53* genotype-dependent trend was observed for urinary metabolites that indicate aminoaciduria (i.e. alanine), lactic aciduria (i.e. lactate) and glycosuria (i.e. glucose). However, *Trp53* genotype had no impact on AAI-DNA adduct levels, as measured by ^32^P-postlabelling, in either target (kidney and bladder) or non-target (liver) tissues, indicating that the underlying mechanisms of p53-related AAI-induced nephrotoxicity cannot be explained by differences in AAI genotoxicity. Performing gas chromatography–mass spectrometry (GC–MS) on kidney tissues showed metabolic pathways affected by AAI treatment, but again *Trp53* status did not clearly impact on such metabolic profiles. We also cultured primary mouse embryonic fibroblasts (MEFs) derived from *Trp53*(+*/*+), *Trp53*(+*/*−) and *Trp53*(−*/*−) mice and exposed them to AAI in vitro (50 µM for up to 48 h). We found that *Trp53* genotype impacted on the expression of NAD(P)H:quinone oxidoreductase (Nqo1), a key enzyme involved in AAI bioactivation. Nqo1 induction was highest in *Trp53*(+*/*+) MEFs and lowest in *Trp53*(−*/*−) MEFs; and it correlated with AAI-DNA adduct formation, with lowest adduct levels being observed in AAI-exposed *Trp53*(−*/*−) MEFs. Overall, our results clearly demonstrate that p53 status impacts on AAI-induced renal injury, but the underlying mechanism(s) involved remain to be further explored. Despite the impact of p53 on AAI bioactivation and DNA damage in vitro, such effects were not observed in vivo.

**Electronic supplementary material:**

The online version of this article (10.1007/s00204-019-02578-4) contains supplementary material, which is available to authorized users.

## Introduction

The p53 transcription factor acts as the “guardian of the genome” by inducing a wide range of tumour suppressive mechanisms (Lane 1992). These include DNA repair, apoptosis, cell cycle arrest and metabolic processes (Vousden and Lane 2007). p53 also acts as a “gatekeeper” by preventing proliferation of damaged cells transiently or permanently, and as a “caretaker” by controlling the repair of genetic alterations within the cell (Kruiswijk et al. 2015; Taneja et al. 2011). Given the wide range of cellular functions regulated by p53, it is inevitable that this protein plays an important role in a wide range of diseases, including cancer (Vousden and Lane 2007). p53 is deregulated in more than 50% of human cancers (Brosh and Rotter 2009; Freed-Pastor and Prives 2012). A disorder known as Li-Fraumeni syndrome, which was shown to be linked to germline mutations in *TP53* and associated with an increased risk of cancer formation, further confirms the critical role played by p53 in tumour suppression (Malkin 2011). Also, *Trp53*(−*/*−) mice develop cancer with complete penetrance (Donehower et al. 1992; Jacks et al. 1994). The deregulation of p53 has also been linked to chemical exposures in the environment. In fact, different environmental carcinogens induce characteristic mutational patterns in *TP53* (Olivier et al. 2010).

**Fig. 1:**
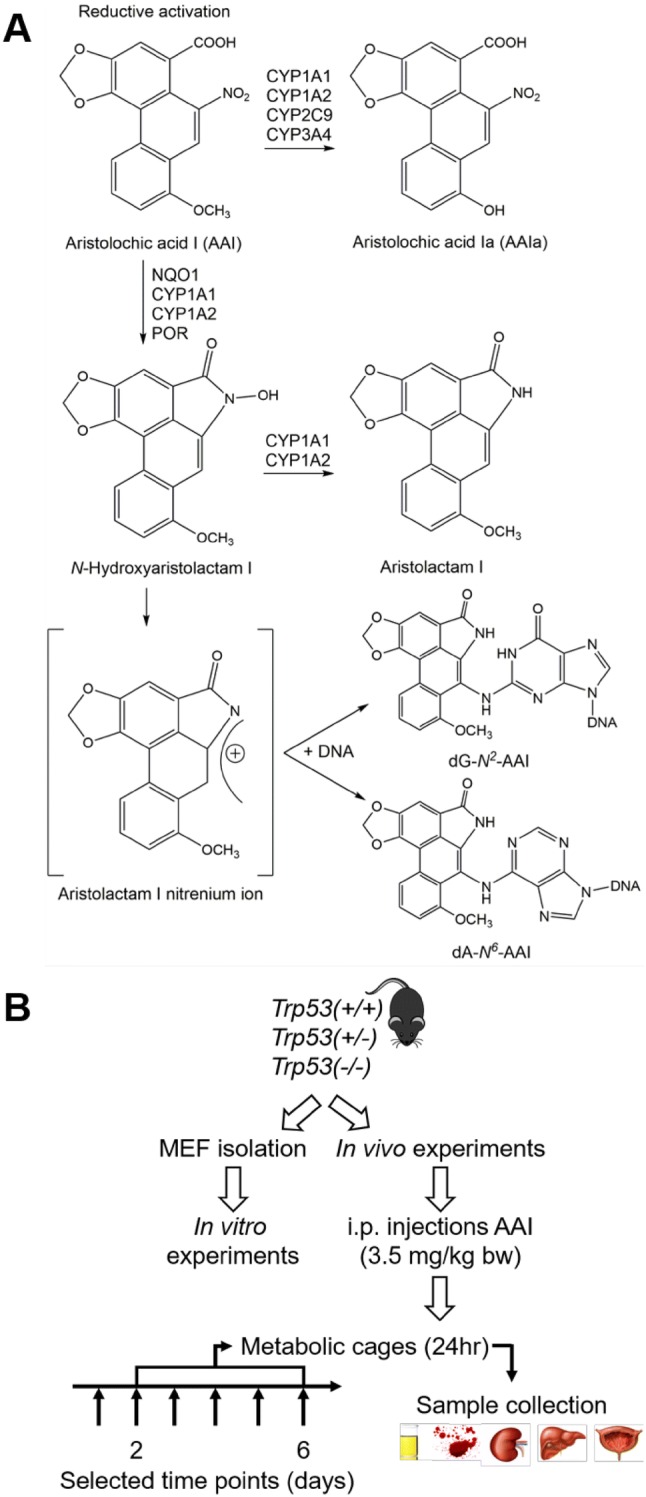
**a** Metabolic activation and detoxication pathways of AAI. CYP, cytochrome P450; dA-*N*^*6*^-AAI, 7-(deoxyadenosin-*N*^*6*^-yl)aristolactam I; dG-*N*^*2*^-AAI, 7-(deoxyguanosin-*N*^*2*^-yl)aristolactam I; NQO, NAD(P)H:quinone oxidoreductase; POR, NADPH:cytochrome P450 oxidoreductase. **b** Schematic representation of experimental design. *Trp53*(+*/*+), *Trp53*(+*/*−) and *Trp53*(−*/*−) mice (*n* = 5/group) were treated with 3.5 mg/kg body weight (bw) AAI by intraperitoneal injection (i.p.) daily for 2 or 6 days. Controls were injected with water only. Mice were placed in metabolic cages on the last day of treatment and sacrificed 24 h later. For in vitro experiments, mouse embryonic fibroblasts (MEFs) were isolated from *Trp53*(+*/*+), *Trp53*(+*/*−) and *Trp53*(−*/*−) mouse embryos

The environmental carcinogen AA is present in *Aristolochia* plants and can be found in medicinal herbal remedies (Gokmen et al. 2013; IARC 2012). The plant extract AA is a mixture of structurally related nitrophenanthrene carboxylic acids, mainly aristolochic acid I (AAI; Fig. 1a) and aristolochic acid II (AAII), with AAI being the major component (Arlt et al. 2002b; Heinrich et al. 2009). AA is the cause of aristolochic acid nephropathy (AAN) and Balkan endemic nephropathy (BEN) (Gokmen et al. 2013; Jadot et al. 2017; Jelakovic et al. 2019; Stiborova et al. 2016). Pathologically, AA-exposed individuals develop extensive interstitial nephropathy, which leads to end-stage renal failure and a high risk of developing upper urinary tract and bladder cancers (Cosyns et al. 1999; Lemy et al. 2008; Nortier et al. 2000). More recently, AA exposure has also been linked to the development of renal cell carcinoma (Hoang et al. 2016; Turesky et al. 2016). The International Agency for Research on Cancer (IARC) has classified AA as carcinogenic to humans (Group 1) (IARC 2012). Since AA is hazardous to human health, many countries have banned *Aristolochia*-containing products from the market (Gokmen et al. 2013). However, the use of AA-containing herbal remedies remains a major concern for public health to date, particularly in Asia (e.g. China and Taiwan) (Grollman 2013).

AA requires metabolic activation before reacting with DNA and exerting its genotoxic effects (Schmeiser et al. 2009). AAI is activated by nitroreduction to generate *N*-hydroxyaristolactam I which can react with DNA to form pre-mutagenic adducts at adenine [i.e. 7-(deoxyadenosin-*N*^6^-yl)-aristolactam I (dA-AAI)] and guanine [i.e. 7-(deoxyguanosin-*N*^2^-yl)-aristolactam I (dG-AAI)] (Fig. 1a) (Stiborova et al. 2017). This nitroreduction is mainly catalysed by NQO1, cytochrome P450 (CYP) 1A1 and 1A2 (CYP1A1 and CYP1A2) and NADPH:cytochrome P450 oxidoreductase enzymes (Fig. 1a) (Stiborova et al. 2013, 2014a). The most abundant DNA adduct detected in AAN and BEN patients is dA-AAI (Arlt et al. 2002a; Nortier et al. 2000; Schmeiser et al. 2012), which leads to characteristic AT to TA transversions in the genomes of AA-exposed tumours (Hoang et al. 2013; Poon et al. 2013). These mutations are also frequently observed in the *TP53* gene (Chen et al. 2012; Grollman et al. 2007), indicating a potential molecular mechanism associated with AA-induced carcinogenesis (Arlt et al. 2011b). Furthermore, AT to TA transversions were also induced in experimental cell culture models, including MEFs derived from Hupki (human *TP53* knock-in) mice, exposed to AA (Kucab et al. 2019; Nedelko et al. 2009; Nik-Zainal et al. 2015). Given the clear link between AAI exposure and p53, it is important to study the role of this gene in AAI tumourigenesis. Previous studies have demonstrated that p53 can impact on carcinogen metabolism (Krais et al. 2016a, b; Willis et al. 2018; Wohak et al. 2016, 2018, 2019). It has also been shown that p53 impacts on the bioactivation of AAI in vitro (Simoes et al. 2008), a phenomenon which requires further investigations to better understand host factors modulating AAI-induced carcinogenesis.

To investigate the role of p53 in AAI-induced nephrotoxicity and DNA damage, *Trp53*(+*/*+), *Trp53*(+*/*−) and *Trp53*(−*/*−) mice were treated with AAI on the basis of a previously established protocol that studies experimental AAN. AAI-induced nephrotoxicity was investigated by performing histological and biochemical analyses on kidney and biofluids, respectively. DNA adduct formation and the activity of AAI metabolising enzymes were measured in kidneys to investigate the impact of p53 function on AAI bioactivation. Metabonomics-based experiments assessed metabolic changes attributable to kidney damage. *Trp53*(+*/*+), *Trp53*(+*/*−) and *Trp53*(−*/*−) MEFs were also employed as in vitro models to further explore the role of p53 in AAI bioactivation.

## Materials and methods

### Carcinogen

Aristolochic acid I (CAS Number: 10190-99-5; AAI; as sodium salt) was isolated as previously reported (Arlt et al. 2017).

### Maintenance of *Trp53***(+*/*+)**, *Trp53***(+*/*−)** and *Trp53***(−*/*−)** mice

*Trp53*(+*/*+), *Trp53*(+*/*−) and *Trp53*(−*/*−) C57BL/6 mice were generated as previously reported (Jacks et al. 1994). *Trp53*(+*/*−) and *Trp53*(−*/*−) mice carry a neomycin cassette that replaces exons 2–6 of the *Trp53* gene, thus eliminating the synthesis of p53 protein (Donehower 2014; Lozano 2009). *Trp53*(−*/*−) mice are viable and their initial development is normal; however, they develop tumours (mostly lymphomas) at 3–6 months of age (Donehower 2014; Lozano 2009). *Trp53*(+*/*−) mice develop sarcomas at approximately 18 months of age (Donehower 2014; Taneja et al. 2011). More information about the *Trp53*^*tm1Tyj*^ mouse strain can be found at www.jax.org/strain/002101. All animal experiments were carried out at King’s College London under licence in accordance with the Animal (Scientific Procedures) Act (1986), as amended by EU Directive 2010/63/EU, and with local ethical approval. Mice were bred at the Biological Services Unit at King’s College London by a *Trp53*(+/−)× *Trp53*(+/−) strategy to maintain the colony and produce *Trp53*(+*/*+), *Trp53*(+*/*−) and *Trp53*(−*/*−) mice and embryos for experiments. All mice were kept under standard conditions with food and water ad libitum.

*Trp53* genotype was determined in mouse pups or embryos by PCR prior to experiments. Ear biopsies were taken from mice at 2–3 weeks of age and DNA was extracted as previously described (Kucab et al. 2015). PCR was performed according to the manufacturer’s instructions using a 2X REDTaq ReadyMix PCR Mix with MgCl_2_ (Sigma-Aldrich). Primers and PCR conditions for an Eppendorf Mastercycler are described in Table S1. PCR products were run on a 2% UltraPure agarose gel (Fig. S1). DNA from *Trp53*(+*/*+) and *Trp53*(−*/*−) mice resulted in one band of 321 bp and 110 bp, respectively; whereas DNA from *Trp53*(+*/*−) mice resulted in two bands, one at 321 bp and the other at 110 bp.

### Treatment of *Trp53***(+*/*+)**, *Trp53***(+*/*−)** and *Trp53***(−*/*−)** mice with AAI

*Trp53*(+*/*+), *Trp53*(+*/*−) and *Trp53*(−*/*−) male mice (9–11 weeks of age; *n* = 5/group) were treated with 3.5 mg/kg bw AAI by intraperitoneal (i.p.) injection daily for 2 or 6 days (Fig. 1b). The dose to inject per mouse was determined by weighing the mice 1 day in advance or on the first day of the experimental protocol. Control mice (*n* = 5/group) were injected with water only. On days 2 or 6, mice were placed individually in metabolic cages to collect urine samples (approximately 1 ml) over 24 h. Mice were killed 24 h after the last treatment; and blood, kidneys, bladder and liver were collected. Tissues were snap frozen in liquid nitrogen and stored at ‒ 80 °C for further analysis. Vertically cut kidneys (whole) were individually placed in 4% paraformaldehyde in phosphate-buffered saline (PBS) at 4 °C. After 24 h, these were washed in 70% ethanol and processed for histology. The urine was centrifuged at 4 °C at 1600 × *g* for 15 min. Urine (50 or 250 µl) was diluted with glycerol (100 or 500 µl) and stored at ‒ 20 °C for quantification of urinary leucine aminopeptidase (LAP) enzymatic activity. The rest of the urine was stored at ‒ 20 °C for creatinine analysis and metabolite analysis by NMR. After collecting the blood (maximum 1 ml) with a syringe containing approximately 100 µl of 0.5 M EDTA, it was centrifuged at 4 °C at 1600 × *g* for 15 min. The upper layer (i.e. serum) was stored at ‒ 20 °C for serum creatinine analysis.

### Histopathology

Formalin-fixed (4% in PBS) and paraffin-embedded sections (4 µM thick) of tissue samples (*n* = 5/group) were attached to poly-l-lysine slides (Sigma-Aldrich). After air drying, the paraffin was removed from the tissue sections with xylene. Colouration by periodic acid-Schiff (PAS) was used for staining kidney samples. Slides were randomised and analysed blindly by optical microscopy. Sections were examined at low (200×) and high (400×) magnification for the presence of cellular injury, dysplasia, neoplasia, inflammation and fibrosis.

Basal glomerular and tubular membranes, and apical membrane brush border of proximal tubules from all PAS-stained kidney tissue samples were carefully examined. Considering the well-preserved structure of the basal membranes, attention was focused on lesions of variable intensity located on the proximal tubular epithelium. A semi-quantitative score of proximal tubular injury was developed and independently assessed by two investigators from the laboratory and one pathologist. The following scoring system was applied: 0—absence of any microscopical abnormality within the renal tissue; 1—proximal tubular injury limited to the loss of the brush border of the apical membrane; 2—necrotic proximal tubules in restricted areas; and 3—large areas of severely necrotic proximal tubules. For each histological sample, a mean quantification was calculated from the three investigators and considered as the final score of injury.

### Biochemical evaluation of renal function

Renal function was determined by measuring creatinine levels by high-performance liquid chromatography (HPLC) in serum samples (*n* = 5/group) as previously reported (Baudoux et al. 2012). A calibration curve with standards containing 13.3–88.4 µmol/l creatinine (Sigma-Aldrich) was set up. The creatinine peak was used to quantify the amount of creatinine (mg/dl) within the serum samples.

LAP enzyme activity was measured with a spectrofluorometric assay in urine samples (*n *= 3/group). Urinary excretion of this enzyme is used as an indicator of the integrity of the proximal tubular brush border (Lebeau et al. 2005). Urine samples (100 µl with glycerol) were diluted 1:30 and 1:60 with 50 mM Tris–HCl buffer (pH 7.6). The substrate leucine-7-amido-4-methyl coumarin (Leu-AMC; Bachem) was incubated with the diluted samples at 37 °C for 60 min. The reaction was terminated by heating the samples at 95 °C for 5 min. Fluorescence of free AMC was measured at 367 nm (excitation) and 440 nm (emission). LAP activity was normalised against urine creatinine (U/g urine creatinine). Creatinine levels in urine (100 µl) were measured using the Jaffé method (Creatinine Diagnostic Kit, Sigma-Aldrich).

### Metabonomics: NMR analysis of urine

Mouse urine samples (*n *= 3/group) prepared for NMR were run on a BrukerAvance 400 spectrometer operating at 400 MHz ^1^H NMR frequency. Briefly, 200 µl of urine was mixed with 340 µl of a buffer (pH 7.4) consisting of 75 mM KH_2_PO_4_ and 15.4 mM sodium azide, and centrifuged at 12,000 rpm (5424R, Eppendorf™) for 30 s at 4 °C. To each sample, 60 µl of a buffer (pH 7.4) consisting of 1.5 M KH_2_PO_4_, 2 mM sodium azide and 6.8 mM 3-(trimethylsilyl)-[2,2,3,3-*d*_4_]-propionic acid sodium salt (TSP-*d*_4_) were added, with TSP-*d*_4_ being used as an internal standard. Following mixing, samples were centrifuged at 12,000 rpm for 5 min at 4 °C. The prepared urine samples were transferred into individual 5 mm NMR tubes (HP507, NORELL^®^), which were capped and stored at 4 °C until analysis. The generated NMR data were analysed as previously reported (Maitre et al. 2017). In brief, NMR spectra were imported into MATLAB^®^ (MathWorks^®^) and normalised against the internal creatinine peak, and results were reported as metabolite levels (i.e. excretion in urine relative to creatinine).

### DNA isolation from mouse tissue

DNA from kidney, liver and bladder tissue (*n *= 4/group) was isolated by a standard phenol–chloroform extraction method. The concentration and purity (260/280 ratio of 1.8-2) of the DNA were measured with a NanoDrop™ 2000 Spectrophotometer.

### DNA adduct analysis by ^32^P-postlabelling

DNA adducts were determined using the nuclease P_1_ enrichment version of the ^32^P-postlabelling assay as previously described (Arlt et al. 2017; Schmeiser et al. 2013). DNA samples (4 µg) were digested with micrococcal nuclease (240 mU, Sigma-Aldrich) and calf spleen phosphodiesterase (60 mU, Calbiochem) for 3 h at 37 °C; and enriched and labelled as reported. For separation by multidirectional thin-layer chromatography (TLC) on polyethyleneimine-cellulose sheets (Macherey–Nagel), the following solvents were used: D1 (1 M sodium phosphate, pH 6.0); D3 (3.5 M lithium formate, 8.5 M urea, pH 4.0); and D4 (0.8 M lithium chloride, 0.5 M Tris, 8.5 M urea, pH 9.0). DNA adducts were visualised by scanning the TLC plates with Instant Imager (Canberra Packard, Dowers Grove) technology. Quantitative analysis was performed as previously described (Phillips and Arlt 2014) and results were expressed as DNA adducts/10^8^ normal nucleotides. AA-DNA adducts were identified using reference compounds as previously described (Schmeiser et al. 1996).

### Preparation of microsomes and cytosols from mouse tissue

Microsomes and cytosols were isolated from untreated (control) and AAI-treated kidney tissues as previously described (Arlt et al. 2008). Microsomal and cytosolic fractions were isolated from pooled tissues (*n* = 5/group), snap frozen in liquid nitrogen and stored at ‒ 80 °C for further analysis. The bicinchoninic acid (BCA) protein assay, with bovine serum albumin (BSA) as a standard, was used to measure protein concentrations in the isolated fractions. Pooled fractions were used for further experiments.

### Immunoblotting

To evaluate the expression of DNA damage response proteins (i.e. p53, p21 and H2ax), whole protein extracts from kidney tissues were prepared as previously described (Krais et al. 2016a). Microsomal and cytosolic fractions were used to determine Cyp1a1 and Nqo1 protein expression, respectively. Western blotting was carried out as previously reported (Kucab et al. 2012). Briefly, protein samples (25 µg and 10 µg of protein for tissue lysates and microsomes or cytosols, respectively) were separated by sodium dodecyl sulphate-polyacrylamide gel electrophoresis using 4–12% gels. Following separation, proteins were transferred onto a nitrocellulose membrane (Bio-Rad) and successful transfer was checked by Ponceau Red (Sigma-Aldrich) staining. After blocking, the following primary antibodies were used overnight at 4 °C: p53 (1:1000; Cell Signalling); p21 (1:2000; BD), H2ax (1:1000; Cell Signalling); Cyp1a1 (1:1000; provided by Dr. Colin Henderson, University of Dundee); and Nqo1 (1:5000, Sigma-Aldrich). Glyceraldehyde phosphate dehydrogenase (Gapdh; 1:25,000; Chemicon) served as a loading control. Following incubations with primary antibodies, blots were incubated with appropriately diluted species-specific horse radish peroxidase-conjugated secondary antibodies (anti-mouse or anti-rabbit; Bio-Rad) for 1 h at room temperature. Proteins were detected by chemiluminescence. Amersham™ ECL™ Western Blotting Detection Reagents (GE Healthcare Life Sciences) was used according to the manufacturer’s instructions and membranes were exposed to Amersham Hyperfilm™ ECL™ (GE Healthcare Life Sciences).

### Enzyme activity assays

7-Ethoxyresorufin *O*-deethylation (EROD) was used to characterise Cyp1a enzyme activity in microsomal fractions (Stiborova et al. 2012). EROD activity was expressed as the amount of resorufin (pmol) produced per concentration of protein (mg/ml) per minute.

Nqo1 activity was measured using menadione (2-methyl-1,4-naphthoquinone) as a substrate as previously reported, and the assay was improved by the addition of cytochrome *c* (Levova et al. 2012). Nqo1 activity was expressed as the amount of reduced cytochrome *c* (nmol) produced per the concentration of protein (mg/ml) per minute.

### RNA isolation from mouse tissue

Total RNA was isolated from kidney tissues (*n* = 5/group) by a modified method based on both TRIzol^®^ (Thermo Fisher Scientific) and RNeasy Mini Kit (QIAGEN) protocols. A portion of tissue (15–35 mg) was placed in a tube containing a steel bead and 1 ml of TRIzol^®^. The tissue was homogenised twice with a TissueLyser II at 25 Hz for 2 min and it was placed at room temperature for 5 min. Following the addition of 200 µl of chloroform, it was centrifuged at 4 °C at 13,000 rpm (5424R, Eppendorf™) for 20 min. The top layer was transferred to a tube and mixed with 350 µl of 70% ethanol. The sample was transferred to an RNeasy Mini Spin column and subsequent RNA isolation steps were performed according to the manufacturer’s instructions. On-column DNase digestion with an RNase-Free DNase Set (QIAGEN) was also performed according to the manufacturer’s instructions. The concentration and purity (260/280 ratio of 2) of the RNA were measured with a NanoDrop™ 2000 Spectrophotometer. The total RNA was stored at ‒ 80 °C for quantitative real-time polymerase chain reaction (qRT-PCR) analysis.

### Gene expression analysis by qRT-PCR

RNA was reverse transcribed into cDNA with a High-Capacity RNA-to-cDNA™ Kit (Thermo Fisher Scientific). qRT-PCR was performed according to the manufacturer’s instructions using a 2X TaqMan™ Gene Expression Master Mix (Thermo Scientific). The Roche Universal Probe Library was used to design intron-spanning assays (i.e. primers and matching probe) for the following NCBI sequences: NM_009992.4 and NM_001136059.2 (*Cyp1a1*); and NM_008706.5 (*Nqo1*). Gene expression was analysed according to the manufacturer’s instructions with a 7500 Fast Real-Time PCR System (Applied Biosystems). Relative gene expression was normalised to the housekeeping gene *Gapdh* (NM_001289726.1) and analysed by the comparative threshold cycle (*C*_t_) method. Results were reported as the fold change in gene expression between the treated and untreated (control) samples (2^−ΔΔCt^ method).

### Metabonomics: GC–MS analysis of mouse tissue

Changes in kidney tissue metabolites (*n *= 5/group) were analysed by GC–MS. Frozen tissues (20–25 mg) were transferred into screw-cap tubes containing glass beads and 800 µl of cold 80% LC–MS CHROMASOLV methanol (Sigma-Aldrich). Tissues were disrupted twice with a Precellys^®^ Evolution homogeniser at 6500 rpm for 20 s. Following centrifugation at 12,000 rpm for 5 min at 4 °C (5424R, Eppendorf™), the supernatant was collected in 2 ml tubes. This metabolite extraction was repeated once more. Obtained samples were dried under nitrogen and stored at ‒ 80 °C for further analysis.

A chloroform (HPLC grade; Sigma-Aldrich)–methanol (2:1) solution (300 µl) was added to each sample on ice and these were vortexed at room temperature at maximum speed for 5 s. LC–MS CHROMASOLV water (300 µl; Sigma-Aldrich) was added to each sample on ice and these were vortexed at room temperature at maximum speed for 5 s. Samples were centrifuged at 12,000 rpm for 10 min at 4 °C. Aqueous and organic phases were transferred into silanised GC–MS glass vials (Agilent Technologies). This dual-phase extraction was repeated once more. Aqueous and organic phases were transferred into the corresponding GC–MS vials. In preparation for derivatisation, 10 µl of myristic acid (Sigma-Aldrich) was added to the samples. Aqueous phases were dried overnight in the following manner: vials were individually sealed with parafilm punctured several times with a needle, placed in liquid nitrogen and a BenchTop Pro with Omnitronics Freeze Dryer (SP Scientific). Organic phases were dried under nitrogen.

Aqueous phases were derivatised by adding 20 µl of a ready-made methoxyamine solution (Thermo Fisher Scientific) to each sample. Samples were incubated at 30 °C at 500 rpm (Thermomixer™, Eppendorf™) for 90 min. Then, 80 µl of *N*-methyl-*N*-(trimethylsilyl)trifluoroacetamide (MSTFA; Thermo Fisher Scientific) was added to each sample and incubated at 37 °C at 500 rpm for 30 min. The liquid in each vial was transferred to a silanised glass insert (Agilent Technologies) within a GC–MS vial for GC–MS analysis.

Organic phases were derivatised by adding 300 µl of a 1:1 methanol-toluene (Sigma-Aldrich) solution and 200 µl of 0.5 M sodium methoxide (in methanol; Sigma-Aldrich) to each sample. Samples were left at room temperature for 1 h. The transmethylation of lipid was stopped by adding 500 µl of 1 M NaCl and 20 µl of concentrated HCl to each sample. Lipid extraction was initiated by adding 500 µl of hexane (Sigma-Aldrich). The top organic layer was transferred to a new GC–MS vial containing magnesium sulphate (Sigma-Aldrich) and the extraction process was repeated once more. Supernatants were combined accordingly, transferred to new GC–MS vials and dried under nitrogen. To derivatise free fatty acids, 40 µl of acetonitrile (Sigma-Aldrich) and 40 µl of MSTFA were added to each sample. Samples were vortexed and spun down briefly before being placed at 37 °C at 350 rpm for 30 min. The liquid in each vial was transferred to a silanised glass insert as described above before commencing GC–MS analysis.

The quality of the technical steps described above was assessed by incorporating a dual-phase extraction blank in both the aqueous and organic phase GC–MS runs. In addition, the quality of the GC–MS runs was determined by injecting quality control (QC) samples (aqueous or organic) after every sixth sample. Such QC samples were created during dual-phase extraction by combining 50 µl or 25 µl of each aqueous or organic phase, respectively. Metabolite chromatograms obtained by GC–MS were analysed against the Automated Mass Spectral Deconvolution and Identification System (AMDIS) database in MATLAB^®^. Spline normalisation was performed against the QC samples in MATLAB^®^. The data were normalised against tissue weight and results were reported as metabolite levels.

### Isolation and culture of *Trp53***(+*/*+)**, *Trp53***(+*/*−)** and *Trp53***(−*/*−)** MEFs

MEFs were isolated from day 13.5 embryos according to a modified protocol as previously described (Kucab et al. 2015). MEFs were genotyped from embryos as described above, and cell stocks were stored in liquid nitrogen. MEFs were grown in Dulbecco’s Modified Eagle’s Medium (DMEM) with high glucose, GlutaMAX™ and pyruvate (Thermo Fisher Scientific), supplemented with 10% foetal bovine serum (FBS) and 100X penicillin–streptomycin. Cells were cultured at 37 °C in 5% CO_2_ and 3% O_2_ for a maximum of 14 days as the cells eventually senesce (Sherr and De Pinho 2000). Following MEF isolation, growth curves were set up for *Trp53*(+*/*+), *Trp53*(+*/*−) and *Trp53*(−*/*−) MEFs to determine appropriate seeding densities for experiments spanning 72 h. *Trp53*(+*/*+) cells doubled over approximately 30 h, whereas *Trp53*(+*/*−) and *Trp53*(−*/*−) cells doubled over approximately 24 h (data not shown). On the basis of these findings, *Trp53*(+*/*+), *Trp53*(+*/*−) and *Trp53*(−*/*−) MEFs were plated at a seeding density of 3 × 10^4^, 2 × 10^4^ and 2 × 10^4^ cells/cm^2^ (24 h), respectively; or 1.5 × 10^4^, 1 × 10^4^ and 1 × 10^4^ cells/cm^2^ (48 h), respectively.

### The impact of *Trp53* status on AAI bioactivation in MEFs

Viability of MEFs after AAI exposure for 24 and 48 h was determined with the crystal violet staining assay as previously described (Kucab et al. 2012). Three concentrations of AAI (10, 50 and 100 µM in water) were tested on the basis of previously published studies (Feldmeyer et al. 2006; Liu et al. 2004; Nedelko et al. 2009). Crystal violet (4[(4-dimethylaminophenyl)-phenyl-methyl]-*N*,*N*-dimethyl-aniline; Sigma-Aldrich) is a dye that stains proteins and DNA. The relative density of the adherent cell culture is a function of the amount of crystal violet staining. Briefly, cell medium was removed from the 96-well cell culture plate and wells were rinsed with 150 µl of PBS. Cells were incubated for 15 min with 30 µl of 0.1% crystal violet per well. Following crystal violet removal, wells were washed with 200 µl of PBS and the plate was allowed to air dry overnight in the dark. At the time of measurement, 100 µl of 50% ethanol was added per well and the absorbance was read at 595 nm on an ELx800 plate reader (BioTek). Cell viability was expressed as the percentage of survival of exposed cells in comparison to the respective control cells. On the basis of the cytotoxicity data, subsequent experiments were carried out solely with 50 µM AAI.

For DNA adduct analysis, MEFs were plated in T75 cm^2^ flasks and exposed to 50 µM AAI for 24 and 48 h. Control cells were exposed to water only. Cell pellets were collected and washed with PBS, and DNA was isolated by a standard phenol–chloroform extraction method. DNA adduct analysis by ^32^P-postlabelling was performed as described above.

To measure protein expression by immunoblotting, MEFs were plated in 6-well cell culture plates and exposed to 50 µM AAI for 24 and 48 h. Control cells were exposed to water only. Cells were collected and washed with PBS, and lysates were prepared as previously reported (Kucab et al. 2019). Western blot analysis was performed as described above.

For gene expression analysis, MEFs were plated in T25 cm^2^ flasks and exposed to 50 µM AAI for 24 and 48 h. Control cells were exposed to water only. Cell pellets were collected and washed with PBS; and RNA was isolated with the RNeasy Mini Kit (QIAGEN) according to the manufacturer’s instructions. qRT-PCR analysis was performed as described above.

### Statistics

The data are presented as mean ± SD, with the sample size dependent on the type of experimental analysis. Statistical analysis was performed with GraphPad Prism 6 software. ANOVA was used to compare three or more groups of data. One-way ANOVA and Tukey’s post hoc test were used when one variable was being compared between three or more groups of data. Two-way ANOVA and Bonferroni’s post hoc test were used when two variables were being compared between three or more groups of data. Results marked with an asterisk (*) indicate comparisons between a treated group to a control group or as otherwise indicated. Results marked with a number sign (#) indicate comparisons to the *Trp53*(+*/*+) group within the treatment group taken into consideration. The following *p* values were used to determine significance: *^(or #)^*p *≤ 0.05, **^(or ##)^*p *≤ 0.01, ***^(or ###)^*p *≤ 0.001, ****^(or ####)^*p *≤ 0.0001.

## Results

### The impact of *Trp53* status on AAI-induced tissue damage

**Fig. 2:**
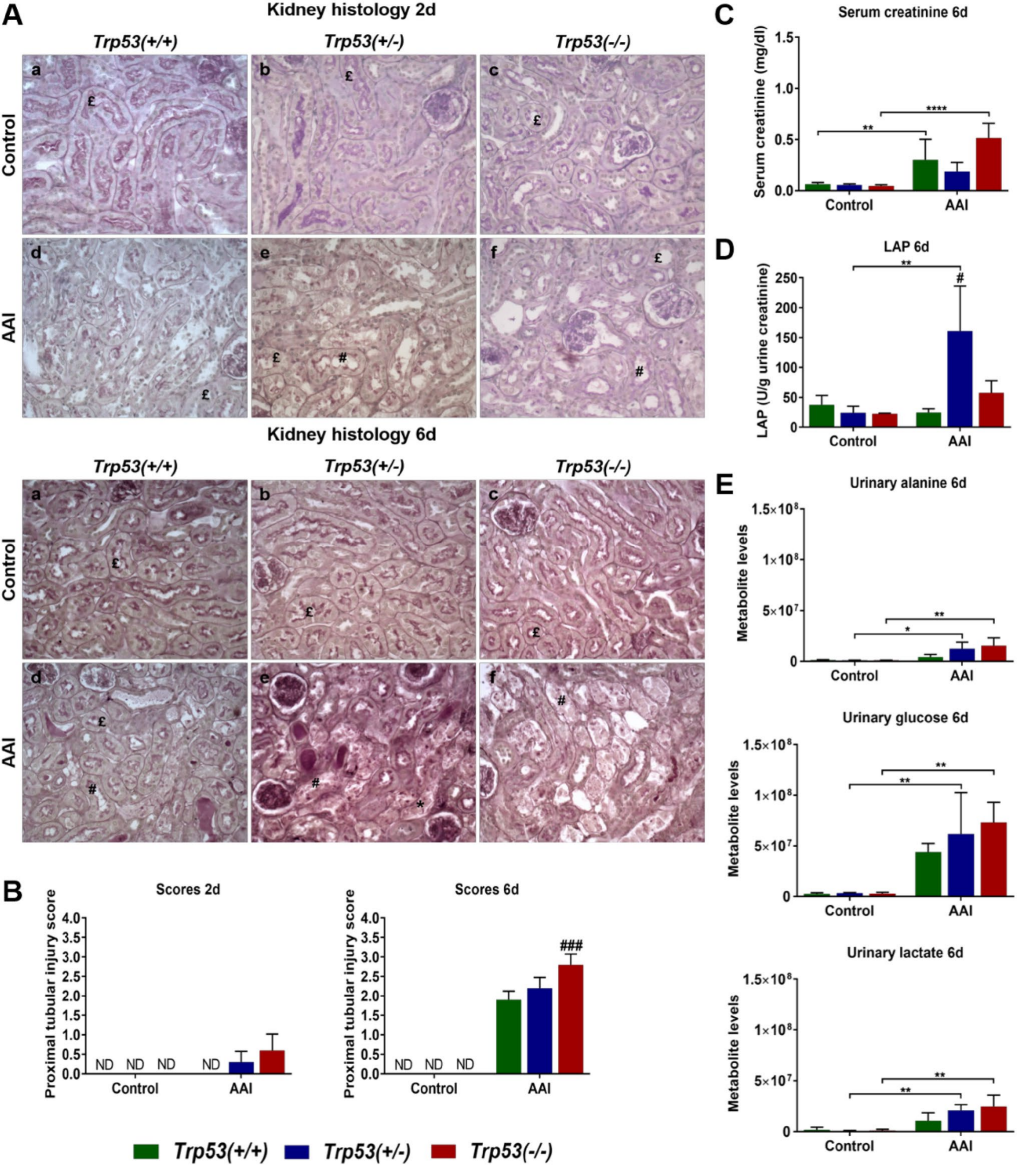
**a** Histopathology of mouse kidneys exposed to AAI for 2 (upper panel) and 6 (lower panel) days. Representative photomicrographs of renal longitudinal sections stained with PAS at a magnification of 400×. Normal proximal tubules (£), injured proximal tubules (#) and necrosis (*) are indicated. **b** Semi-quantitative proximal tubular injury score at 2 (left panel) and 6 (right panel) days. Scores were determined on PAS-stained kidney sections (*n* = 5 mice/group). *ND* not detected. **c** Serum creatinine levels in AAI-treated mice (6 days). Serum creatinine (mg/dl) levels were determined by HPLC analysis (*n* = 5 mice/group). **d** LAP enzyme activity in AAI-treated mice (6 days). LAP activity (U/g urine creatinine) was determined using a spectrofluorometric assay (*n* = 3 mice/group). **e** Urinary metabolite levels in AAI-treated mice (6 days). Alanine (upper panel), glucose (middle panel) and lactate (lower panel) levels (arbitrary units) in urine were measured by NMR (*n* = 3 mice/group). Results are presented as mean ± SD. Statistical analysis was performed by two-way ANOVA and Bonferroni’s post hoc test (*p ≤ 0.05, **p ≤ 0.01, ****p ≤ 0.0001, comparison as indicated); and by one-way ANOVA and Tukey’s post hoc test [^#^*p *≤ 0.05, ^###^*p *≤ 0.001, in comparison to *Trp53*(+*/*+) within that treatment group]

To investigate the impact of *Trp53* on AAI-induced tissue damage and toxicity, kidney tissues obtained from AAI-treated *Trp53*(+*/*+), *Trp53*(+*/*−) and *Trp53*(−*/*−) mice were analysed by histopathology. Representative photomicrographs (400 × magnification) for kidneys are shown in Fig. 2a. Discrepancies in colours between photomicrographs were simply due to staining or the settings of the optical microscope. PAS staining delineates both the apical brush border and basal membrane of the proximal tubular epithelium (shown in dark pink in the photomicrographs).

At both 2 and 6 days, the proximal tubules in untreated (control) *Trp53*(+*/*+), *Trp53*(+*/*−) and *Trp53*(−*/*−) kidneys were found to be of normal structure. After treatment with AAI for 2 days, *Trp53*(+*/*+) kidneys were normal. However, at the same time point, there was an indication of mild or mild-to-moderate proximal tubular injury in AAI-exposed *Trp53*(+*/*−) kidneys. This injury was characterised by local detachment of the brush border membrane. However, proximal tubular necrosis was not found. In *Trp53*(−*/*−) kidneys, moderate-to-severe tubular injury was observed. In some, but not all *Trp53*(−*/*−) kidneys, areas of proximal tubular necrosis were found. After treatment with AAI for 6 days, a gradient of intensity in proximal tubular lesions from *Trp53*(+*/*+) to *Trp53*(+*/*−) kidneys and then to *Trp53*(−*/*−) kidneys was evident. Proximal tubular injury was isolated in *Trp53*(+*/*+) kidneys and it consisted of the disappearance of the brush border membrane. Renal injury in *Trp53*(+*/*−) mice was characterised by severe lesions of necrosis. In these mice, the lumen of the proximal tubules was obstructed by debris; however, oedema or inflammatory infiltrate were not present. In *Trp53*(−*/*−) kidneys, proximal tubular injury and necrosis were both extensive and severe. Atrophic proximal tubules were also noted. Such tubules are unable to regenerate the brush border membrane and appear pseudo-dilated. At a lower magnification (Fig. S2; 200× magnification), areas with both damaged and intact proximal tubules were observed in AAI-exposed *Trp53*(+*/*−) and *Trp53*(−*/*−) kidney sections. A lower number of proximal tubules, however, kept their normal structure in *Trp53*(−*/*−) kidneys.

Given the renal injury in AAI-treated *Trp53*(+*/*+), *Trp53*(+*/*−) and *Trp53*(−*/*−) mice at both 2 and 6 days, kidney tissue sections were semi-quantitatively scored (Fig. 2b). As expected, a score of 0 was given to untreated (control) *Trp53*(+*/*+), *Trp53*(+*/*−) and *Trp53*(−*/*−) kidneys at both 2 and 6 days. The scores attributed to AAI-exposed *Trp53*(+*/*+), *Trp53*(+*/*−) and *Trp53*(−*/*−) kidneys were reflective of the histopathology noted in Fig. 2a. More precisely, the proximal tubular injury was most severe in *Trp53*(−*/*−) kidneys at both 2 and 6 days. The differences in renal injury between *Trp53*(−*/*−) (i.e. score of 2.8) and *Trp53*(+*/*+) (i.e. score of 1.9) kidneys were most significant at 6 days. Overall, histopathological scores revealed that wild-type (WT) *Trp53* protects from AAI-induced proximal tubular damage.

To monitor the toxicity of AAI in mice, body weights of *Trp53*(+*/*+), *Trp53*(+*/*−) and *Trp53*(−*/*−), mice treated with AAI for 2 or 6 days were measured (Fig. S3). Whilst AAI treatment for 2 days had no effect on weight, a slight decrease in weight was observed for *Trp53*(+*/*+), *Trp53*(+*/*−) and *Trp53*(−*/*−) mice treated with AAI for 6 days. However, this change in weight (from day 0 to day 6) was not statistically significant.

Since kidney injury was most prominent after 6 days of AAI treatment, further investigations related to the role of *Trp53* in AAI-induced nephrotoxicity and DNA damage solely focused on this time point.

### The impact of *Trp53* status on markers of AAI-induced nephrotoxicity

To further investigate the impact of *Trp53* on AAI-induced nephrotoxicity, creatinine levels were measured by HPLC analysis in serum. As expected, serum creatinine levels were low in untreated (control) mice but increased after AAI treatment (Fig. 2c). However, this increase was only significant for AAI-treated *Trp53*(+*/*+) and *Trp53*(−*/*−) mice, with serum creatinine levels being the highest in *Trp53*(−*/*−) mice.

Urinary LAP enzyme activity was used as a marker to assess renal proximal tubular damage. LAP enzyme activity was low in urine of untreated (control) mice. Urinary LAP activity only significantly increased in AAI-treated *Trp53*(+*/*−) mice in comparison to the respective control and AAI-treated *Trp53*(+*/*+) mice (Fig. 2d). In AAI-treated *Trp53*(−*/*−) mice, LAP activity appeared to be slightly higher relative to AAI-treated *Trp53*(+*/*+) mice, but this effect was not statistically significant.

A metabonomic approach was also used to further investigate markers of AAI-induced renal injury in urine. NMR spectra for nine metabolites were selected for analysis. These included alanine, α-ketoglutarate, citrate, glucose, lactate, succinate, trimethylamine, formate and fumarate. Interestingly, alanine, glucose and lactate levels increased after AAI treatment (Fig. 2e). These changes were solely significant in AAI-treated *Trp53*(+*/*−) and *Trp53*(−*/*−) mice in comparison to their respective controls. There also seemed to be an emerging pattern in which levels of these urinary metabolites (i.e. alanine, glucose and lactate) increased in a *Trp53* genotype-dependent manner, with the lowest levels in *Trp53*(+*/*+) mice and the highest levels in *Trp53*(−*/*−) mice. However, these changes were not statistically significant. Interestingly, succinate and trimethylamine levels decreased in urine (Fig. S4), with succinate levels significantly lower in AAI-treated *Trp53*(+*/*+) and *Trp53*(−*/*−) mice compared to controls, and trimethylamine levels significantly lower in AAI-treated *Trp53*(+*/*+), *Trp53*(+*/*−) and *Trp53*(−*/*−) mice. Moreover, urinary trimethylamine levels were significantly higher in AAI-treated *Trp53*(+*/*−) mice relative to AAI-treated *Trp53*(+*/*+) mice. In contrast, no significant changes in α-ketoglutarate, citrate, fumarate and formate levels were observed (Fig. S4).

### The impact of *Trp53* status on AAI-induced DNA damage

**Fig. 3:**
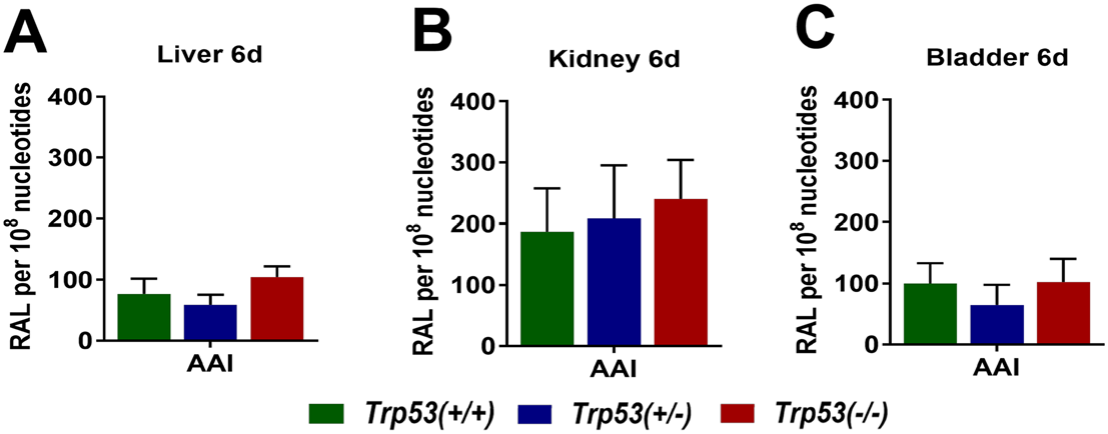
AAI-DNA adduct formation (RAL, relative adduct labelling) in **a** liver, **b** kidney and **c** bladder at 6 days was determined by the nuclease P1-enrichment version of the ^32^P-postlabelling method. Results are presented as mean ± SD (*n* = 4 mice/group)

The adduct pattern induced by AAI in liver, kidney and bladder was qualitatively similar in *Trp53*(+*/*+), *Trp53*(+*/*−) and *Trp53*(−*/*−) mice. The pattern consisted of two major adduct spots, previously identified (Schmeiser et al. 2009) as dA-AAI (spot 1) and dG-AAI (spot 2) (Fig. S5a). In addition, one minor adduct was detected, previously identified (Schmeiser et al. 2009) as 7-(deoxyadenosin-*N*^6^-yl)-aristolactam II (dA-AAII; spot 3) (Fig. S5a). These adducts (i.e. dA-AAI, dG-AAI and dA-AAII) have been found in urothelial tissue of AAN patients (Nortier et al. 2000; Schmeiser et al. 2014; Stiborova et al. 2017). No DNA adducts were detected in untreated (control) tissues (data not shown). AAI-induced DNA adduct levels were the highest in kidney, with levels being ~ 2.5-fold higher in kidney relative to both bladder and liver (Fig. 3). However, no differences were observed in DNA adduct formation between *Trp53*(+*/*+), *Trp53*(+*/*−) and *Trp53*(−*/*−) mice.

### The impact of *Trp53* status on AAI-induced DDR

**Fig. 4:**
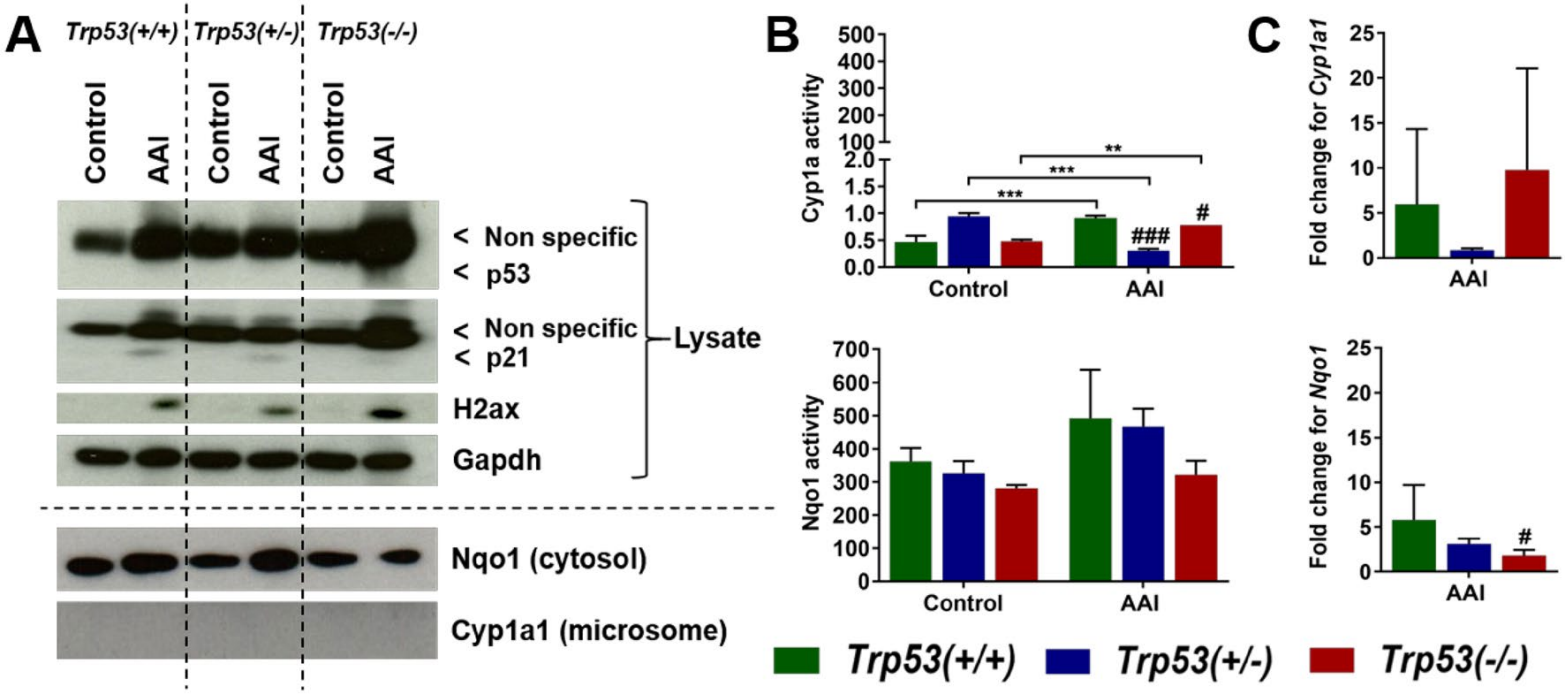
**a** Protein expression in AAI-exposed mouse kidneys. Western blot analysis of p53, p21 and H2ax expression in kidney tissue from AAI-treated (6 days) *Trp53*(+*/*+), *Trp53*(+*/*−) and *Trp53*(−*/*−) mice. Nqo1 and Cyp1a1 expression was investigated in AAI-exposed (6 days) kidney cytosols and microsomes, respectively. Gapdh expression was used as a loading control. Representative images of Western blots are shown; at least duplicate analyses from independent experiments were performed. **b** Enzyme activity in AAI-exposed mouse kidneys. Cyp1a activity at 6 days (upper panel) was determined by the EROD assay. Results are presented as mean ± SD derived from independent in vitro measurements (*n* = 3/group). Nqo1 activity at 6 days (lower panel) was determined by the NQO1 assay. Results are presented as mean ± SD derived from independent in vitro measurements (*n* = 3/group). **c** Gene expression in AAI-exposed mouse kidneys. The fold change for *Cyp1a1* (upper panel) and *Nqo1* (lower panel) is the fold change in expression relative to the control for each genotype. Gene expression was determined by qRT-PCR and the 2^−ΔΔCt^ method (*n* = 5 mice/group). Statistical analysis was performed by two-way ANOVA and Bonferroni’s post hoc test (**p ≤ 0.01, ***p ≤ 0.001, ****p ≤ 0.0001, comparison as indicated); and by one-way ANOVA and Tukey’s post hoc test (^#^*p *≤ 0.05, ^##^*p *≤ 0.01, ^###^*p *≤ 0.001, ^####^*p *≤ 0.0001, in comparison to *Trp53*(+*/*+) within that treatment group)

The expression of DDR proteins was investigated in kidney tissue lysates. p53 expression was not detected in *Trp53*(+*/*+), *Trp53*(+*/*−) and *Trp53*(−*/*−) kidneys (Fig. 4a). Observed bands in the range of interest were unspecific and close to the expected p53 bands. However, film exposure for shorter and longer periods of time ascertained that p53 is undetectable. p21 expression was also not detected in untreated (control) kidneys, whereas weak induction was noticeable in AAI-exposed kidneys (Fig. 4a). As a marker of DNA damage, particularly the formation of double-strand breaks (Dickey et al. 2009), expression of histone H2A family member X (H2ax) was investigated. H2ax was induced in AAI-exposed kidneys, whereas such effect was not found in respective controls (Fig. 4a). H2ax induction seemed to be higher in AAI-exposed *Trp53*(−*/*−) kidneys in comparison to AAI-exposed *Trp53*(+*/*+) and *Trp53*(+*/*−) kidneys.

### The impact of *Trp53* status on the expression of AAI metabolising enzymes

Nqo1 and Cyp1a1 protein expression was investigated in kidney cytosols and microsomes, respectively. Nqo1 was induced in AAI-exposed *Trp53*(+*/*+) and *Trp53*(+*/*−) kidney cytosols relative to controls, whereas no such induction was observed in AAI-exposed *Trp53*(−*/*−) kidney cytosols (Fig. 4a). Cyp1a1 was not detected at the protein level in kidney microsomes (Fig. 4a). In contrast, Cyp1a enzyme activity was detectable in kidney microsomes, but it was relatively low (Fig. 4b). Basal Cyp1a activity was *Trp53* genotype dependent and significantly altered by AAI treatment. Cyp1a enzyme activity was significantly lower in AAI-exposed *Trp53*(+*/*−) and *Trp53*(−*/*−) kidneys relative to AAI-exposed *Trp53*(+*/*+) kidneys. It also significantly decreased in AAI-exposed *Trp53*(+*/*−) kidneys in comparison to controls, whilst the activity of Cyp1a in AAI-exposed *Trp53*(+*/*+) and *Trp53*(−*/*−) kidneys significantly increased relative to controls. Nqo1 enzyme activity was measured in kidney cytosols (Fig. 4b). In general, Nqo1 enzyme activity was relatively high. Nqo1 activity appeared to be dependent on *Trp53* genotype and treatment, with the lowest activity in AAI-exposed *Trp53*(−*/*−) kidneys relative to AAI-exposed *Trp53*(+*/*+) kidneys, but observed changes were not statistically significant. *Cyp1a1* and *Nqo1* expression was also investigated in kidney tissues at the gene level by qRT-PCR (Fig. 4c). No significant induction in *Cyp1a1* expression was observed after AAI treatment. In contrast, *Nqo1* expression was significantly lower in AAI-exposed *Trp53*(−*/*−) kidneys relative to AAI-exposed *Trp53*(+*/*+) kidneys, thus reflecting findings at the protein level in AAI-exposed kidney cytosols.

### The impact of *Trp53* status on kidney tissue metabolite profiles after AAI treatment

**Fig. 5:**
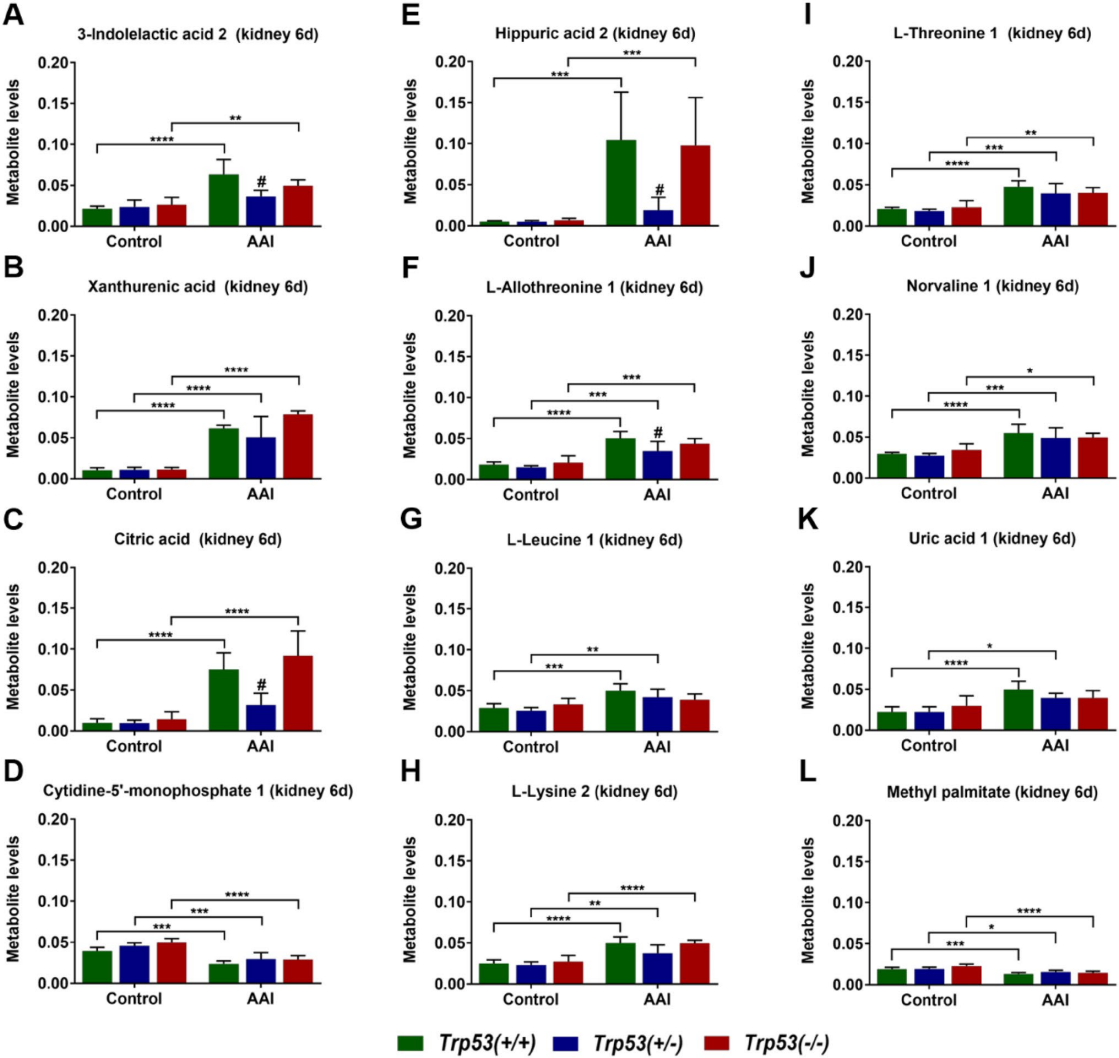
Metabolite levels in kidney tissues of AAI-treated mice (6 days). **a** 3-indolelactic acid 2, **b** xanthurenic acid, **c** citric acid, **d** cytidine-5′-monophosphate 1, **e** hippuric acid 2, **f**l-allothreonine 1, **g**l-leucine 1, **h**l-lysine 2, **i**l-threonine 1, **j** norvaline 1, **k** uric acid 1 and (**l**) methyl palmitate levels (arbitrary units) in kidney tissues were measured by GC–MS. Results are presented as mean ± SD (*n* = 5 mice/group). Statistical analysis was performed by two-way ANOVA and Bonferroni’s post hoc test (*p ≤ 0.05, **p ≤ 0.01, ***p ≤ 0.001, ****p ≤ 0.0001, comparison as indicated); and by one-way ANOVA and Tukey’s post hoc test (^#^*p *≤ 0.05, in comparison to *Trp53*(+*/*+) within that treatment group)

An additional metabonomic method, GC–MS, was used to detect changes in metabolite levels in kidneys of AAI-treated *Trp53*(+*/*+), *Trp53*(+*/*−) and *Trp53*(−*/*−) mice. A total of 72 and 25 metabolites were detected in aqueous (Table S2) and organic (Table S3) phases, respectively. For most metabolites, levels were highly variable between genotype or treatment groups and unaffected by AAI treatment. However, 11 metabolites detected in the aqueous phase of AAI-exposed kidneys displayed significant changes following AAI treatment (Fig. 5): 3-indolelactic acid 2, xanthurenic acid, citric acid, cytidine-5′-monophosphate 1, hippuric acid 2, l-allothreonine 1, l-leucine 1, l-lysine 2, l-threonine 1, norvaline 1 and uric acid 1. For ten out of these 11 metabolites, levels significantly increased after AAI treatment in most genotype groups. Interestingly, 3-indolelactic acid 2 (Fig. 5a), citric acid (Fig. 5c), hippuric acid 2 (Fig. 5e) and l-allothreonine 1 (Fig. 5f) levels were significantly lower in AAI-exposed *Trp53*(+*/*−) kidneys compared to AAI-exposed *Trp53*(+*/*+) kidneys. A different trend was observed for cytidine-5′-monophosphate 1 (Fig. 5d). More precisely, the levels of this metabolite significantly decreased in AAI-exposed *Trp53*(+*/*+), *Trp53*(+*/*−) and *Trp53*(−*/*−) kidneys compared to respective controls. Only one metabolite from the organic phase of AAI-exposed kidneys was affected by AAI treatment. More precisely, methyl palmitate levels significantly decreased in AAI-exposed *Trp53*(+*/*+), *Trp53*(+*/*−) and *Trp53*(−*/*−) kidneys (Fig. 5l). Overall, AAI treatment impacted on the level of certain metabolites in kidney tissue. Despite observing a pattern in metabolite levels for AAI-exposed *Trp53*(+*/*−) kidneys, *Trp53* genotype did not majorly impact on tissue metabolite levels after AAI treatment.

### The impact of *Trp53* status on AAI bioactivation and DNA damage in MEFs

**Fig. 6:**
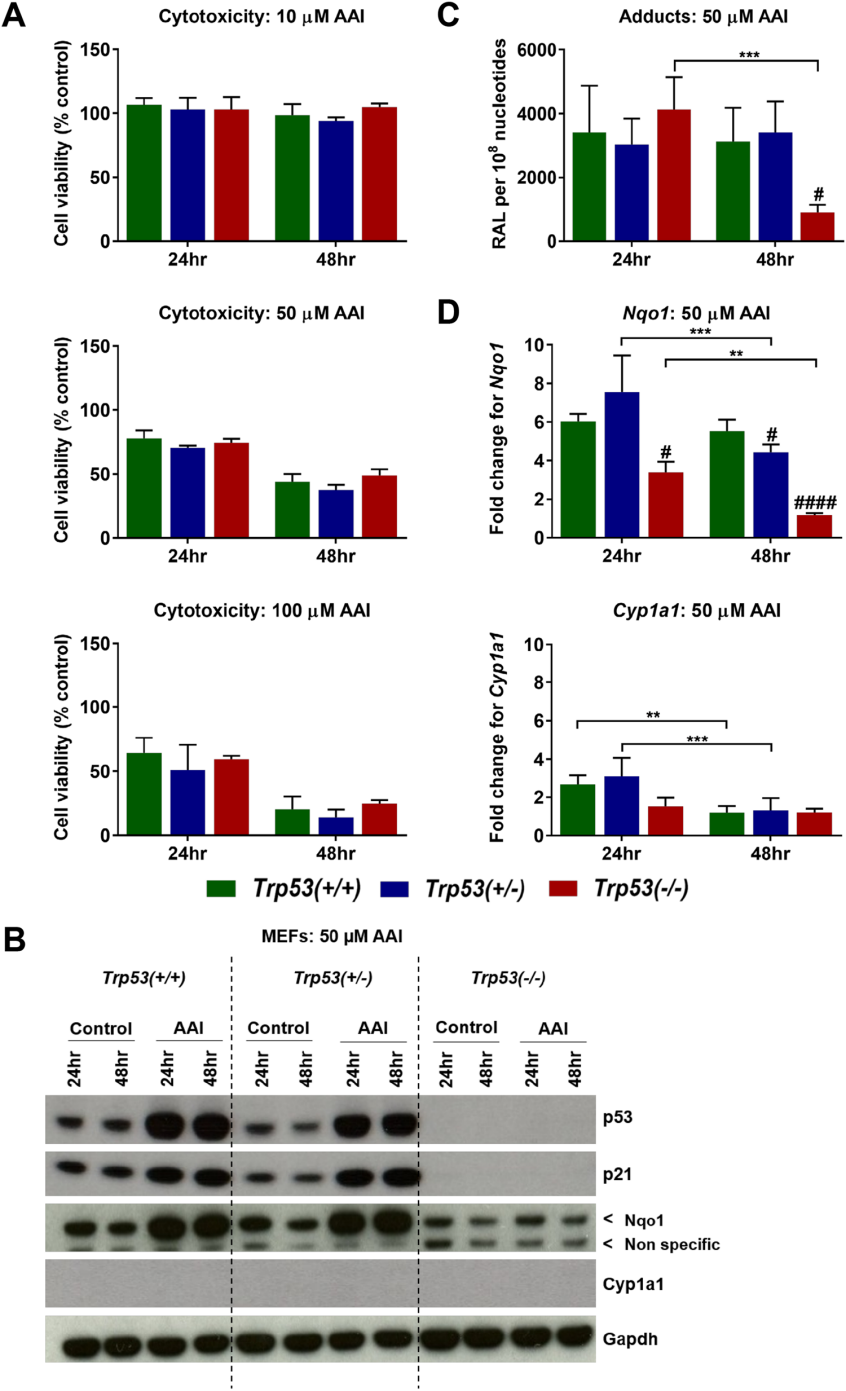
**a** Cell viability in AAI-exposed MEFs. *Trp53*(+*/*+), *Trp53*(+*/*−) and *Trp53*(−*/*−) MEFs were exposed to 10 µM (upper panel), 50 µM (middle panel) and 100 µM (lower panel) AAI for 24 and 48 h. Controls were exposed to water only. Cell viability was assessed with the crystal violet assay following 24 and 48 h exposure to AAI. Results are presented as mean ± SD (*n* = 3/group) derived from independent experiments with cells from different stocks from the same embryo. **b** Protein expression in AAI-exposed MEFs. Western blot analysis of p53, p21, Nqo1 and Cyp1a1 expression at 24 and 48 h (50 µM AAI). Gapdh expression was used as a loading control. Representative images of Western blots are shown; at least duplicate analyses from independent experiments were performed. **c** DNA adduct formation in AAI-exposed MEFs. AAI-DNA adduct formation (RAL, relative adduct labelling; 50 µM AAI) was determined by the nuclease P1-enrichment version of the ^32^P-postlabelling method. Results are presented as mean ± SD (*n* = 4/group) derived from independent experiments with cells from different stocks from the same embryo. **d** Gene *e*xpression in AAI-exposed MEFs. The fold change (50 µM AAI) for *Nqo1* (upper panel) and *Cyp1a1* (lower panel) is the fold change in expression relative to the water control for each cell type. Gene expression was determined by qRT-PCR and the 2^−ΔΔCt^ method. Results are presented as mean ± SD (*n* = 4/group) derived from independent experiments with cells from different stocks from the same embryo. Statistical analysis was performed by two-way ANOVA and Bonferroni’s post hoc test (**p  ≤ 0.01, ***p  ≤ 0.001, comparison as indicated); and by one-way ANOVA and Tukey’s post hoc test [^#^*p *≤ 0.05, ^####^*p *≤ 0.0001, in comparison to *Trp53*(+*/*+) within that exposure group]

To determine cell viability following AAI exposure, *Trp53*(+*/*+), *Trp53*(+*/*−) and *Trp53*(−*/*−) MEFs were exposed to 10, 50 and 100 µM AAI for 24 and 48 h (Fig. 6a). Exposure to 10 µM AAI for 24 and 48 h did not lead to cytotoxicity in MEFs. Cell viability was only slightly affected in MEFs at 50 µM AAI for 24 h (> 60% viability), but cell survival decreased at this concentration at 48 h (< 60% viability). Exposure to 100 µM AAI was highly cytotoxic (≤ 60% viability) at both 24 and 48 h in MEFs. *Trp53* genotype did not significantly impact on cell viability at any of the tested AAI concentrations and time points. To study the effects of AAI on DNA damage in MEFs, 50 µM AAI was selected for further experiments.

As expected, p53 and p21 were expressed in *Trp53*(+*/*+) and *Trp53*(+*/*−) MEFs, but not in *Trp53*(−*/*−) MEFs (Fig. 6b). p53 was highly induced in AAI-exposed *Trp53*(+*/*−) MEFs. p53 protein was also highly induced in AAI-exposed *Trp53*(+*/*−) MEFs, but at lower levels in comparison to *Trp53*(+*/*+) MEFs. p21 was also induced over 48 h in AAI-exposed *Trp53*(+*/*+) and *Trp53*(+*/*−) MEFs, with no striking differences in p21 levels between *Trp53*(+*/*+) and *Trp53*(+*/*−) MEFs. AAI treatment induced DNA adducts and the pattern observed in *Trp53*(+*/*+), *Trp53*(+*/*−) and *Trp53*(−*/*−) MEFs was qualitatively similar. The pattern consisted of two major adduct spots, previously identified (Schmeiser et al. 2009) as dA-AAI (spot 1) and dG-AAI (spot 2) (Fig. S5b). The same AAI-DNA adducts were formed in vivo (Fig. S5a). No DNA adducts were detected in untreated (control) MEFs (data not shown). AAI-DNA adduct levels were not significantly different between *Trp53*(+*/*+), *Trp53*(+*/*−) and *Trp53*(−*/*−) MEFs at 24 h (Fig. 6c). However, at 48 h, adduct levels were significantly lower in *Trp53*(−*/*−) MEFs in comparison to *Trp53*(+*/*+) MEFs after AAI treatment. In addition, adduct levels in *Trp53*(−*/*−) MEFs exposed to AAI for 48 h were significantly lower in comparison to those exposed for 24 h.

Expression of Nqo1 and Cyp1a1 in MEFs was investigated both at the protein (Fig. 6b) and gene (Fig. 6d) levels. Interestingly, unlike for AAI-exposed *Trp53*(−*/*−) MEFs, Nqo1 was highly induced at both 24 and 48 h in AAI-exposed *Trp53*(+*/*+) and *Trp53*(+*/*+) MEFs (Fig. 6b). At 48 h, these findings correlated with the observed adduct levels. Overall, *Trp53* genotype affected Nqo1 induction in AAI-exposed MEFs. More strikingly, *Nqo1* expression was significantly lower in *Trp53*(−*/*−) MEFs than in *Trp53*(+*/*+) MEFs following AAI exposure at both 24 and 48 h (Fig. 6d). Cyp1a1 was not detected at the protein level in MEFs (Fig. 6b). Experiments on MEFs isolated from different embryos supported this observation (data not shown). *Cyp1a1* was expressed, but not significantly altered, between *Trp53*(+*/*+), *Trp53*(+*/*−) and *Trp53*(−*/*−) MEFs at both 24 and 48 h (Fig. 6d). However, *Cyp1a1* levels significantly decreased from 24 to 48 h in both *Trp53*(+*/*+) and *Trp53*(+*/*−) MEFs.

## Discussion

### The impact of *Trp53* status on AAI-induced tissue damage

In experimental animals, treatment with higher doses of AA leads to necrosis in proximal tubules, a histopathological feature also seen in AAN patients. For example, daily i.p. treatment with 5 mg/kg bw AA over 8 days (Baudoux et al. 2012) or oral treatment with 5 mg/kg bw AAI over 21 days (Arlt et al. 2011b) led to necrosis in proximal tubules in mice. Renal necrosis was also observed in another rodent model, in which rats were treated with subcutaneous injections of either 10 mg/kg bw AA for 10 days (Debelle et al. 2002) or 15 mg/kg bw AA for 5 days (Pozdzik et al. 2016). In the present study, experimental AAN was studied in mice as previously described (Baudoux et al. 2012), but *Trp53*(+*/*+), *Trp53*(+*/*−) and *Trp53*(−*/*−) mice were treated with a daily i.p. dose of 3.5 mg/kg bw AAI, instead of 5 mg/kg bw, in order to minimise necrotic lesions in kidneys (Nortier et al., unpublished observation). The chosen time points (2 and 6 days) in the present study were also based on previous work (Baudoux et al. 2012) and selected to monitor acute AAI-induced nephrotoxicity over time. As performed previously (Baudoux et al. 2012), only male mice were used in the present study. It was not possible to conduct experiments on female mice because female *Trp53*(−*/*−) mice appeared to have high mortality soon after birth. It is noteworthy that previous work on mice, which were treated with 5 mg/kg bw AA for 21 days, led to tumour formation in the forestomach, kidneys, lungs, uterus and lymphoid organs within 56 weeks (Mengs et al. 1982). In the present study, AAI-exposed kidneys appeared macroscopically abnormal, especially at 6 days and in *Trp53*(−*/*−) mice (data not shown). In addition, AAI-treated *Trp53*(−*/*−) mice presented early signs of sickness (e.g. lethargy, loss of appetite, impaired movement) towards day 6. Thus, AAI treatment for more than 6 days at the selected dose was not considered for ethical reasons. Moreover, at 6 days, *Trp53*(−*/*−) mice tended to excrete urine that was lighter in colour, indicating some form of diuresis.

The kidneys act as filters by removing toxic waste from the blood through glomerular filtration (Scott and Quaggin 2015). The membranes of epithelial cells (e.g. proximal tubular cells) within the kidney both reabsorb beneficial and secrete unwanted metabolites. Hence, proximal tubular cells come into close contact with xenobiotics, including nephrotoxic compounds (Nicholson et al. 2002). AA primarily targets proximal tubules in human kidney (Nortier and Vanherweghem 2002). Experimentally, AA uptake was confirmed in vitro (i.e. by opossum kidney proximal tubular cells) (Lebeau et al. 2001). It was shown that organic anion transporters mediate uptake of AA into proximal tubular cells and thereby participate in renal cell damage (Bakhiya et al. 2009; Dickman et al. 2011; Xue et al. 2011). The histopathology findings in the present study clearly showed that prolonged AAI treatment (i.e. 6 days) damages kidneys, especially renal proximal tubular cells. Similar histopathological damage was also shown in previous rodent studies (Arlt et al. 2011b; Baudoux et al. 2012; Debelle et al. 2002, 2003; Pozdzik et al. 2016). Apart from proximal tubular necrosis, both atrophy and fibrosis with a lymphocytic infiltrate were present (Baudoux et al. 2012). In addition, the latter study showed that the brush border of proximal tubules presented with disruptions and detachment. Other studies also demonstrated that glomeruli remain intact after AA treatment (Debelle et al. 2003), which is in accordance with the findings in the present study.

In the present study, proximal tubular damage depended on *Trp53* status. More precisely, renal injury was more severe in AAI-treated *Trp53*(−*/*−) mice relative to *Trp53*(+*/*+) and *Trp53*(+*/*−) mice. Interestingly, these findings contrast with a previous study in which *Trp53*(+*/*+) mice treated daily with 10 mg/kg bw AA over 3 days presented with more severe kidney damage in comparison to *Trp53*(−*/*−) mice (Zhou et al. 2010). Moreover, the study by Zhou et al. (2010) demonstrated that AA-exposed *Trp53*(+*/*+) mouse kidney tissues present with high levels of apoptosis. Based on in vitro experiments in rat kidney tubular epithelial NRK-52E cells, the authors concluded that AA-induced apoptosis is activated by STAT3 (signal transducer and activator of transcription 3)-mediated phosphorylation of *Trp53* (Zhou et al. 2010). Several reasons could explain why an opposite trend was observed in the study by Zhou et al. (2010). For example, a different mouse strain (i.e. C57BL/6-Tyrc) was used, and the mutant *Trp53* mice were tagged with a gene that differentiates *Trp53* genotypes according to coat colour. Biological outcomes could also be affected by the fact that Zhou et al. (2010) used the natural plant extract AA, which contains a mixture of AAI (65%) and AAII (27%), whereas pure AAI was used in the present study. This should not majorly affect findings as AAI, rather than AAII, is considered to be the nephrotoxic component of AA (Sato et al. 2004; Shibutani et al. 2007). However, it can be speculated that the observed differences are related to dose. Zhou et al. (2010) used a higher daily dose of AA (10 mg/kg bw) compared to the present study (3.5 mg/kg bw AAI daily). Since p53 plays a major role in apoptotic pathways (Vousden 2000), it could be that p53 protects from AAI-induced cellular stress at lower doses (e.g. through a DNA repair mechanism), whereas higher doses activate the pro-apoptotic functions of p53. Romanov et al. (2015) examined mechanisms of AA-induced apoptosis in cultured human renal epithelial HK-2 cells. Their study showed that a low concentration of AAI (i.e. 4 µM) induces activation of DNA damage signalling pathways and cell cycle arrest, whereas a high concentration of AAI (i.e. 40 µM) leads to more severe damage and cell death, partly by apoptosis (Romanov et al. 2015). Further investigations could examine the extent of apoptosis in kidney tissues exposed to AAI in more detail. Determining the extent of proximal tubule apoptosis was previously carried out in AA-treated rats (Pozdzik et al. 2008), but such investigations were beyond the scope of the present study.

Previous studies showed that rodents lose weight after a prolonged treatment with AA (Debelle et al. 2002, 2003; Shibutani et al. 2007). Drastic weight loss occurred after a chronic treatment (i.e. 35 days) with AA (up to 10 mg/kg bw daily) (Debelle et al. 2002). Weight loss appeared to be noticeable in *Trp53*(+*/*+), *Trp53*(+*/*−) and *Trp53*(−*/*−) mice treated with AAI for 6 days, but this effect was not statistically significant. Similar findings were reported in mice treated with 2.5 mg/kg bw AAI, where body weight loss was not prominent until after day 5–6, but noticeable weight loss (i.e. 5–6 g) was observed by day 9 (Shibutani et al. 2007). Interestingly, the latter study reported that kidneys appeared pale at day 10. The kidneys collected in the present study also showed macroscopic changes at day 6.

### The impact of *Trp53* status on biochemical markers of nephrotoxicity after AAI treatment

Renal damage can be monitored by different means. For example, low molecular weight proteins can be measured in urine, since damaged proximal tubular cells are unable to reabsorb these (Lebeau et al. 2005). Low molecular weight proteins found in urine include β_2_-microglobulin, cystatin C, Clara cell protein, retinal-binding protein and α_1_-microglobulin (Kabanda et al. 1995). Such proteins can be released from different areas of the proximal tubular epithelium after AAI-induced nephrotoxicity (Lebeau et al. 2005). LAP is an example of an enzyme that is used experimentally to monitor AA-induced renal damage and it tends to be excreted from the brush border membrane of the proximal tubule. In the present study, LAP activity increased at 6 days, particularly in urine of AAI-treated *Trp53*(+*/*−) mice. This can be explained by the fact that AAI-exposed *Trp53*(+*/*−) proximal tubules presented with detachment of the brush border membrane and necrosis. LAP activity in urine of AAI-treated *Trp53*(−*/*−) mice also seemed to be higher. However, the activity of the enzyme was not as high as for AAI-exposed *Trp53*(+*/*−) urine, and this change was not statistically significant. This can be explained by the fact that atrophic *Trp53*(−*/*−) proximal tubules are unable to regenerate the brush border membrane and, consequently, cannot secrete LAP as *Trp53*(+*/*−) cells. Other studies also showed that LAP activity increases following AA treatment. For example, LAP activity increased in the urine of rats treated daily with 10 mg/kg bw AA for 3 and 7 days (Lebeau et al. 2005). However, LAP activity decreased after day 7 in AA-treated rats. Other studies in rats also showed that LAP activity increases up to a certain period of AA treatment (Debelle et al. 2002, 2003), suggesting that LAP activity is a marker of acute AA-induced nephrotoxicity.

Measuring creatinine in serum is another method with which to monitor renal function (Debelle et al. 2003). Creatinine results from creatinine phosphate metabolism and it is kept at a constant rate through glomerular filtration (Duquesne et al. 2017; Klawitter et al. 2010). If such filtration is affected through damage, creatinine accumulates in the blood and decreases in the urine. Previous studies in fact showed that treatment with AA increases serum creatinine levels in rats (Debelle et al. 2002, 2003; Lebeau et al. 2005). In the present study, creatinine levels increased in *Trp53*(+*/*+), *Trp53*(+*/*−) and *Trp53*(−*/*−) serum after 6 days of AAI treatment, thus reflecting the kidney injury observed by histopathology. There was a trend to observe higher creatinine levels in serum obtained from AAI-treated *Trp53*(−*/*−) mice, which is in line with the increased kidney damage observed in such mice.

### The impact of *Trp53* status on the metabolome after AAI treatment

Metabonomics is a branch of “-omics” technologies whereby changes in the metabolome of an organism can be determined by analysing tissues and biofluids (e.g. urine, blood, cell medium) (Keun and Athersuch 2011; Nicholson et al. 2002). NMR is advantageous as it is simple, non-destructive and non-invasive, and it can be used to analyse complex mixtures (Beckonert et al. 2007; Chatham and Blackband 2001; Keun and Athersuch 2011). A disadvantage of NMR, however, is that it is less sensitive than other metabonomic methods, such as GC–MS (Chatham and Blackband 2001; Lenz and Wilson 2007). The advantage of the latter method is that global metabolic profiles can be compared to commercial databases (Lenz and Wilson 2007).

Interestingly, urine-related NMR spectra reflect the areas of the kidney that are subject to injury (Nicholson et al. 2002). For example, damage to proximal tubules (in the renal cortex) is linked to abnormal levels of creatinine, glucose, alanine and valine in urine. However, abnormal lactate and alanine levels in urine are characteristic of glomerular-related damage. Moreover, xenobiotics that target the renal medulla give rise to a different metabolic fingerprint to those that damage the renal cortex (Neild et al. 1997). These conclusions are based on rodent studies in which animals were treated with different toxicants that target the kidney in a region-specific manner. For example, a rat study of mercury(II) chloride, a proximal tubular toxicant, revealed aminoaciduria (e.g. alanine, glutamine, valine) and lactic aciduria (Gartland et al. 1988).

Under normal physiological conditions, amino acids (e.g. alanine), glucose and lactate are reabsorbed throughout the proximal tubule (Bellomo 2002; Curthoys and Moe 2014). Interestingly, glucose reabsorption is attributed to the action of glucose transporters (e.g. glucose transporter 1) located in proximal tubular membranes (Rahmoune et al. 2005). Hence, if such tubules and their membranes are damaged, glucose will leak into the urine. This also applies to amino acids and lactate. The latter metabolite is in fact associated with proximal tubular damage and necrosis (Hauet et al. 2000). In the present study, alanine, glucose and lactate levels were strikingly higher in the urine of *Trp53*(+*/*+), *Trp53*(+*/*−) and *Trp53*(−*/*−) mice after 6 days of AAI treatment, indicating aminoaciduria, glycosuria and lactic aciduria, respectively. This is in line with recent findings in which urine from AAI-treated rats (75 mg/kg bw) demonstrated a similar metabolic fingerprint (Duquesne et al. 2017). In addition, high levels of glucose were previously found in rats treated with 10 mg/kg bw AA (Debelle et al. 2002). Lactic aciduria could potentially indicate the induction of anaerobic respiration in response to a lack of glucose or glomerular damage in the kidney (Duquesne et al. 2017). However, in the present study, the glomeruli in AA-exposed kidneys seemed normal.

In the present study, succinate decreased in the urine of AAI-treated *Trp53*(+*/*+), *Trp53*(+*/*−) and *Trp53*(−*/*−) mice. This metabolite is a citric acid cycle intermediate and its reduction was noted previously in AAI-treated rats (Duquesne et al. 2017). Its reduction in urine could be explained by a dampened citric acid cycle. Trimethylamine is produced by intestinal bacteria in humans (e.g. from choline) and can be ingested through the diet (Zeisel and Warrier 2017). The reduction of trimethylamine in the urine of AAI-treated *Trp53*(+*/*+), *Trp53*(+*/*−) and *Trp53*(−*/*−) mice could thus be an indication of lower food consumption. In fact, mice treated with AAI for 6 days displayed early signs of sickness, which is normally associated with a decrease in appetite. Previous work on rats noted a decrease in food consumption following AAI treatment relative to controls (Duquesne et al. 2017). However, in the present study, insignificant changes in body weight after 6 days of AAI treatment could indicate that a decrease in trimethylamine levels is unrelated to dietary changes.

Overall, certain urinary metabolites (e.g. glucose) clearly acted as biomarkers for AAI-induced nephrotoxicity. Despite the lack of a statistically significant *Trp53* genotype-dependent response on urinary metabolite levels in AAI-treated mice, it can be speculated that some sort of a pattern emerges when looking closely at all the investigated metabolites. More precisely, urinary metabolite levels increased from AAI-treated *Trp53*(+*/*+) to *Trp53*(−*/*−) mice (especially for alanine, glucose and lactate). This observation would reflect the more severe injury observed in AAI-treated *Trp53*(−*/*−) mice compared to AAI-treated *Trp53*(+*/*+) mice. A larger mouse study could possibly be required to observe clear differences between genotypes (Halouska et al. 2013; Marshall and Powers 2017).

AAI-induced changes in the metabolome were also investigated in AAI-exposed *Trp53*(+*/*+), *Trp53*(+*/*−) and *Trp53*(−*/*−) kidneys by GC–MS. Overall, findings showed that AAI treatment alters the quantity of certain metabolites in kidney tissues. However, *Trp53* status did not majorly contribute to metabolite level alterations. Two metabolites that play a role in metabolising dietary tryptophan, 3-indolelactic acid 2 and xanthurenic acid, increased in all kidney samples after AAI treatment. Both indole and xanthurenic acid play a role in the kynurenine pathway of tryptophan metabolism (Badawy 2017). Interestingly, previous work on AA-exposed human renal proximal tubular HK-2 cells showed that AA affects tryptophan metabolism (Liu et al. 2016). In the present study, amino acid levels also increased in AAI-exposed kidneys. These amino acids included l-allothreonine 1, l-leucine 1, l-lysine 2, l-threonine 1 and norvaline 1, thus indicating some sort of shift in amino acid metabolism after AAI treatment. Previous studies on rat urine demonstrated that AA treatment leads to increased amino acid levels (Hu et al. 2017; Ni et al. 2007). Citric acid also increased strikingly in AAI-treated kidneys. It could be that citric acid levels in tissue are associated with changes in acidosis and alkalosis (Curthoys and Moe 2014; Relman 1972). Interestingly, the effect on citric acid levels was more prominent in tissues than in urine after AAI treatment. However, a definite explanation cannot be given as to why citric acid levels increased in tissue, since a significant increase in other citric acid cycle intermediates was not noted in AAI-exposed kidneys. Uric acid is a product of nucleotide metabolism and hippuric acid plays a role in both tryptophan and phenylalanine metabolism (Hu et al. 2017). Both these metabolites increased in AAI-exposed kidneys. These were previously identified as biomarkers of AA exposure in rodents (Hu et al. 2017; Zhao et al. 2015a). It has been previously shown that kidney injury unrelated to AAI exposure can lead to changes in nucleotide metabolism, especially purine metabolism (Wei et al. 2014). However, a decrease in the levels of the nucleotide cytidine-5′-monophosphate 1 in AAI-exposed kidneys indicated that AAI treatment impacts on pyrimidine metabolism. Further investigations would be required to determine the significance of AAI-induced perturbations in nucleotide metabolism in vivo. AA exposure can perturb lipid and fatty acid metabolism, which can in turn be linked to inflammation (Lou et al. 2011; Zhao et al. 2015b). In the present study, methyl palmitate decreased in AAI-exposed kidneys. This does not concur with a previous GC–MS study in which palmitate levels increased in kidneys isolated from rats treated with AA (10 and 20 mg/kg bw daily for 7 days) (Lou et al. 2011). It could be that an opposite trend in methyl palmitate levels was observed in the present study because the dosage of AAI was lower and signs of inflammation were not observed by histopathology. Nonetheless, a decrease in methyl palmitate levels indicated a shift in fatty acid metabolism.

Overall, GC–MS analysis of kidney tissues did not fully mirror the findings obtained with urine by NMR. However, metabolite perturbations in kidneys revealed that AAI-treated mice exhibit changes in metabolites that play a role in tryptophan, amino acid, nucleotide and fatty acid metabolism.

### The impact of *Trp53* status on AAI bioactivation and DNA damage in vivo

To elucidate the role of *Trp53* in AAI bioactivation in vivo, DNA damage (i.e. AAI-DNA adduct formation) was investigated in livers (non-target organs) as well as kidneys and bladders (both target organs). Liver and kidney in particular play important roles in AAI metabolism (Stiborova et al. 2013, 2014a). In the present study, the formation of DNA adducts was highest in kidneys, which is in agreement with a number of studies in which mice were treated acutely or sub-chronically with AAI (Arlt et al. 2011b, 2017; Shibutani et al. 2007; Stiborova et al. 2012). However, *Trp53* genotype had no impact on AAI-DNA adduct formation in vivo despite the impact of *Trp53* genotype on AAI-induced renal injury. These in vivo results do not support in vitro findings where *TP53* genotype clearly impacted on AAI-induced DNA adduct formation (Simoes et al. 2008). However, *Trp53* could, however, still impact on the bioactivation of AAI through another mechanism.

Expression of p53 protein was not detected in untreated (control) and AAI-exposed *Trp53*(+*/*+), *Trp53*(+*/*−) and *Trp53*(−*/*−) kidneys. The findings are in line with previous work in which p53 was not detected in untreated (control) and AAI-exposed (5 mg/kg bw AAI daily for 3, 12 and 21 days) *TP53*(+*/*+) Hupki mouse kidneys (Arlt et al. 2011b). p53 protein is also not detected in normal kidney tissue in humans (HPA 2018), but p53 induction has been observed in urothelial tumours of AAN patients (Cosyns et al. 1999). On the other hand, AAI exposure resulted in the induction of p53 in *TP53*(+*/*+) HCT116 cells (Simoes et al. 2008), suggesting that AAI exposure may impact differently on the cellular accumulation of p53 in vitro and in vivo. Other DDR proteins were also investigated. p21, a major target of p53 (Kruiswijk et al. 2015), was weakly induced in *Trp53*(+*/*+), *Trp53*(+*/*−) and *Trp53*(−*/*−) kidneys. This is in accordance with a previous study in which AAI-exposed (5 mg/kg bw daily) *TP53*(+*/*+) Hupki kidneys exhibited p21 induction only after 12 and 21 days, but not 3 days of treatment (Arlt et al. 2011a). Nevertheless, the observed p21 induction was higher at 12 days than what was found in this study at 6 days, potentially indicating that prolonged treatment with AAI is required to detect significant changes in p21 expression. AAI has also been shown to induce the formation of DNA strand breaks in cells and H2ax is used as a marker for double-strand breaks (Chen et al. 2010; Dickey et al. 2009). In the present study, H2ax induction was highest in kidneys of AAI-treated *Trp53*(−*/*−) mice, thus going in hand with the proximal tubular injury observed in such tissues.

AAI is activated and detoxified by several enzymes, which include NQO1 and CYP1A1 (Stiborova et al. 2013, 2014a). We utilised multiple approaches (i.e. protein expression, enzyme activity and gene expression) to study the potential impact of *Trp53* on AAI bioactivation in the kidney. NQO1 is the most potent cytosolic enzyme capable of catalysing the bioactivation of AAI (Barta et al. 2014; Stiborova et al. 2011, 2014b). Here, Nqo1 protein expression increased in AAI-exposed *Trp53*(+*/*+) and *Trp53*(+*/*−) kidney cytosols. This induction of Nqo1 protein in kidney tissue has been previously shown in mice treated with AAI (Arlt et al. 2011b; Barta et al. 2014). In the present study, it could be argued that Nqo1 expression was unaffected by AAI treatment in *Trp53*(−*/*−) kidney cytosols, thus indicating a *Trp53*-dependent response on Nqo1 expression. A similar effect was observed for *Nqo1* gene expression, whereas this response was less pronounced when measuring Nqo1 enzyme activity. Collectively, these findings indicate that any *Trp53* genotype-dependent impact on Nqo1 activity does not lead to a substantial alteration of AAI bioactivation; hence, no differences were observed in DNA adduct formation between AAI-exposed *Trp53*(+*/*+), *Trp53*(+*/*−) and *Trp53*(−*/*−) kidneys.

Enzymes of the CYP1A family (i.e. CYP1A1 and CYP1A2) play an important role in AAI metabolism due to their dual role in AAI activation and detoxication (Levova et al. 2011). Furthermore, previous studies showed that Cyp1a1 and Cyp1a2 are expressed in proximal tubular cells (Schaaf et al. 2001). The responses on Cyp1a1/2 in the present study were complex, which could be linked to the fact that the observed effects were small or absent. The lack of Cyp1a1 protein expression could be explained by the fact that the Cyp1a1 antibody used may not be sensitive enough to detect low Cyp1a1 levels. Cyp1a activity was *Trp53* genotype-dependent in AAI-exposed kidneys, but generally responses differed between untreated (control) and AAI-exposed *Trp53*(+*/*+), *Trp53*(+*/*−) and *Trp53*(−*/*−) kidney microsomes. Overall, Cyp1a activity in kidneys was low, indicating that this enzyme might not contribute to AAI metabolism in these tissues. This was further confirmed by investigating *Cyp1a1* expression in kidney tissue. Again, these findings are in accord with the fact that no differences were observed in DNA adduct formation between AAI-exposed *Trp53*(+*/*+), *Trp53*(+*/*−) and *Trp53*(−*/*−) kidneys.

### The impact of *Trp53* status on AAI bioactivation in vitro

The lack of influence of *Trp53* genotype on AAI-induced DNA adduct formation in vivo does not support previous in vitro findings where *TP53* genotype clearly impacted on AAI bioactivation (i.e. AAI-DNA adduct formation) in human cells (Simoes et al. 2008). To further test the hypothesis that p53 influences AAI bioactivation in vitro, the genotoxic effects of AAI were investigated in *Trp53*(+*/*+), *Trp53*(+*/*−) and *Trp53*(−*/*−) MEFs. Cell viability in these MEFs decreased in a concentration-dependent manner. This was shown in other studies using MEFs and confirmed the previously characterised cytotoxicity of AAI (Krais et al. 2015). AAI-DNA adduct levels in *Trp53*(+*/*+), *Trp53*(+*/*−) and *Trp53*(−*/*−) MEFs suggested that AAI bioactivation might be *Trp53*-dependent in this mouse in vitro model. More precisely, at 48 h, DNA adducts were lower in AAI-exposed *Trp53*(−*/*−) MEFs relative to *Trp53*(+*/*+) MEFs. Interestingly, this finding reflects observations in AAI-exposed HCT116 cells (i.e. higher adduct levels in *TP53*(+*/*+) cells relative to *TP53*(−*/*−) cells), although the underlying mechanism has not yet been elucidated in these cells (Simoes et al. 2008).

Nqo1 expression was investigated in AAI-exposed *Trp53*(+*/*+), *Trp53*(+*/*−) and *Trp53*(−*/*−) MEFs. The induction of Nqo1 at the protein level was found to be *Trp53*-dependent, with the highest induction occurring in *Trp53*(+*/*+) and *Trp53*(+*/*−) MEFs. No changes were observed in AAI-exposed *Trp53*(−*/*−) MEFs. The mRNA data for *Nqo1* also demonstrated *Trp53*-dependent changes (particularly at 48 h). As shown at the protein level, *Nqo1* expression was at its lowest in *Trp53*(−*/*−) MEFs. Since the expression of Nqo1 correlated with AAI-DNA adduct levels, a novel role for *Trp53* in AAI bioactivation was identified. The findings for Nqo1 also mirror those found in vivo.

## Conclusions

The in vivo findings in this study indicate that WT *Trp53* protects against AAI-induced nephrotoxicity. Measuring markers of nephrotoxicity (i.e. LAP and serum creatinine) and urinary metabolites did not show a clear-cut *Trp53* genotype-dependent response. The histopathological and biochemical observations are supported by the metabonomic measurements, where a consistent *Trp53* genotype-dependent trend was observed for a number of urinary metabolites, but with high inter-individual variability. Performing GC–MS analysis on kidney tissues showed metabolic pathways that may be affected by AAI treatment, but *Trp53* status did not clearly impact on such metabolic profiles. Nevertheless, discovering a *Trp53* genotype-dependent response on proximal tubular damage prompted further studies to explore the mechanisms by which *Trp53* affects AAI-induced DNA damage. The findings underlined the DNA damaging properties of AAI but showed that *Trp53* status has no impact on AAI-induced DNA adduct formation. *Trp53* genotype did not significantly impact on Cyp1a and Nqo1 in AAI-exposed kidneys, indicating that p53 function does not modulate AAI metabolism in vivo. As *Trp53* status clearly impacted on AAI-induced nephrotoxicity, the underlying mechanism(s) cannot be explained by differences in AAI genotoxicity and remains to be further explored.

The in vitro study indicated that *Trp53* impacts on Nqo1 expression after AAI exposure and supported the in vivo findings. Potential mechanisms by which *Trp53* regulates or influences the induction of Nqo1 remain to be further investigated. In this context, it is noteworthy that polymorphisms in the human *NQO1* gene have been reported to be important in BEN patients and the NQO1*2 (C609T) genotype predisposed BEN patients to the development of urothelial cancer (Stiborova et al. 2016; Toncheva et al. 2004). Given the importance of *TP53* mutations in cancers arising from AA exposures (Chen et al. 2012; Grollman et al. 2007; Lord et al. 2004), it would also be crucial to further investigate the role of p53 in AAI bioactivation with other in vitro models. These could include human HCT116 cells with differing *TP53* genotypes (including *TP53* mutants) (Wohak et al. 2016) and MEFs bearing *TP53* mutations relevant to AAI carcinogenesis (Odell et al. 2013). Mouse models carrying specific *TP53* mutations frequently observed in human tumours are also available to further explore the impact of mutant *TP53* on AAI-induced nephrotoxicity and DNA damage (Song et al. 2007).

## Electronic supplementary material

Below is the link to the electronic supplementary material.

Supplementary material 1 (DOCX 7569 kb)
