# Supplementary material for: The impact of p53 on aristolochic acid I-induced nephrotoxicity and DNA damage in vivo and in vitro
Source: Arch Toxicol. 2019 Oct 10;93(11):3345–66. doi: 10.1007/s00204-019-02578-4 (PMC6823306; doi:10.1007/s00204-019-02578-4)
Supplement: Supplementary file 1 — Supplementary material 1 (DOCX 7569 kb) [file 204_2019_2578_MOESM1_ESM.docx]

**Electronic Supplementary Material, Archives of Toxicology**

**The impact of p53 on aristolochic acid I-induced nephrotoxicity and DNA damage *in vivo* and *in vitro***

Mateja Sborchia^1^, Eric G. De Prez^2^, Marie-Hélène Antoine^2^, Lucie Bienfait^3^, Radek Indra^4^, Gabriel Valbuena^5^, David H. Phillips^1^, Joëlle L. Nortier^2^, Marie Stiborová^4^, Hector C. Keun^5^ and Volker M. Arlt^1^

^1^Department of Analytical, Environmental and Forensic Sciences, MRC-PHE Centre for Environment and Health, King’s College London, London, SE1 9NH, United Kingdom

^2^Laboratory of Experimental Nephrology, Department of Experimental Biochemistry, Faculty of Medicine, Université libre de Bruxelles, 1070, Brussels, Belgium

^3^Department of Pathology, Erasme University Hospital, 1070, Brussels, Belgium

^4^Department of Biochemistry, Faculty of Science, Charles University Prague, 128 40, Prague, Czech Republic

^5^Department of Surgery and Cancer, Faculty of Medicine, Imperial College London, London, W12 0NN, United Kingdom

Corresponding author: Dr. Volker M. Arlt, Department of Analytical, Environmental and Forensic Sciences, King’s College London, London, SE1 9NH, UK. E-mail: [volker.arlt@kcl.ac.uk](mailto:volker.arlt@kcl.ac.uk). Telephone: +44 (0)20 7848 3781


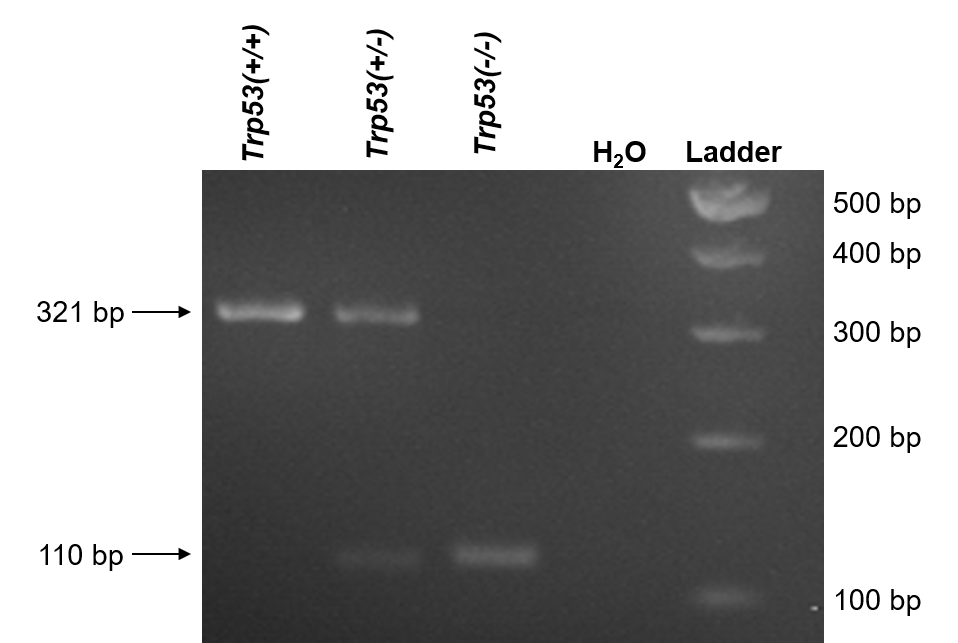


**Fig. S1** Example of a *Trp53(+/+)*, *Trp53(+/-)* and *Trp53(-/-)* mouse genotyping PCR. H_2_O indicates the negative control. bp, base pairs.


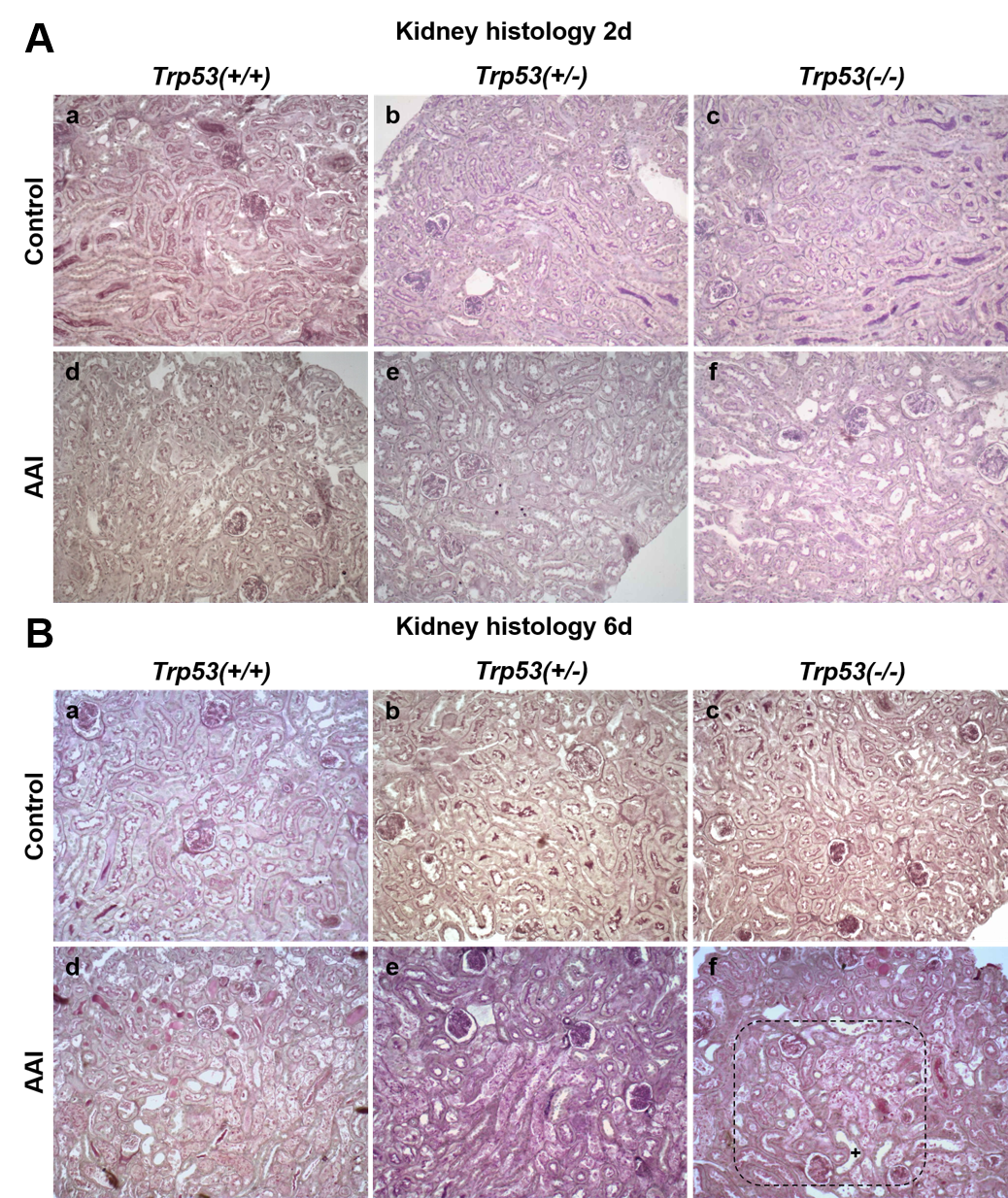


**Fig. S2** Histopathology of mouse kidneys exposed to AAI for (**a**) 2 and (**b**) 6 days. Representative photomicrographs of renal longitudinal sections stained with PAS at a magnification of 200x. Areas of proximal tubular injury (---) and atrophic proximal tubules (+) are indicated.

**Fig. S3** Mouse weights after (**a**) 2 and (**b**) 6 days of AAI treatment. Mouse body weights were measured one day in advance or on the first day of the experimental protocol (i.e. 0d); and on the day of sacrifice (i.e. 6d). Mouse weight (g; grams) is presented as mean ± SD (*n* = 5 mice/group).


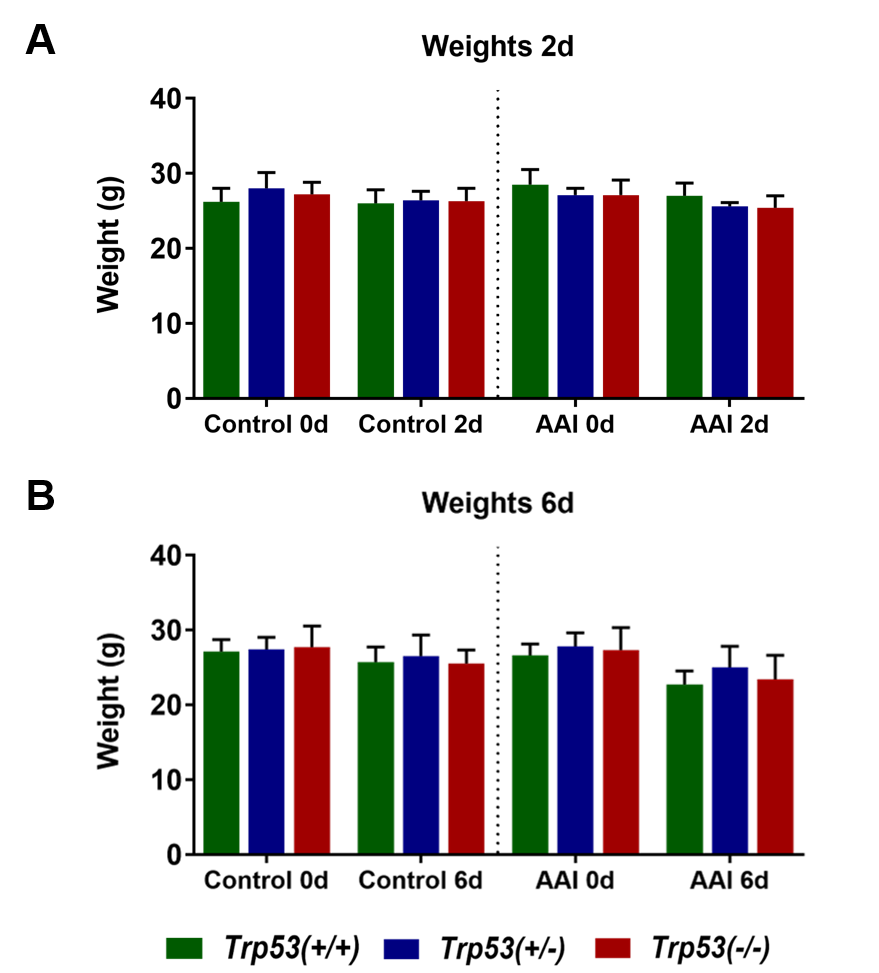


**Fig. S4** Urinary metabolite levels in AAI-treated mice (6 days). (**a**) α-ketoglutarate, (**b**) citrate, (**c**) succinate, (**d**) trimethylamine, (**e**) formate and (**f**) fumarate levels (arbitrary units) in urine were measured by NMR. Note the different scales for (**e**) and (**f**). Results are presented as mean ± SD (*n* = 3 mice/group). Statistical analysis was performed by two-way ANOVA and Bonferroni’s post-hoc test (**p*≤0.05, ***p*≤0.01, *****p*≤0.0001, comparison as indicated); and by one-way ANOVA and Tukey’s post-hoc test (^#^*p*≤0.05, in comparison to *Trp53(+/+)* within that treatment group).


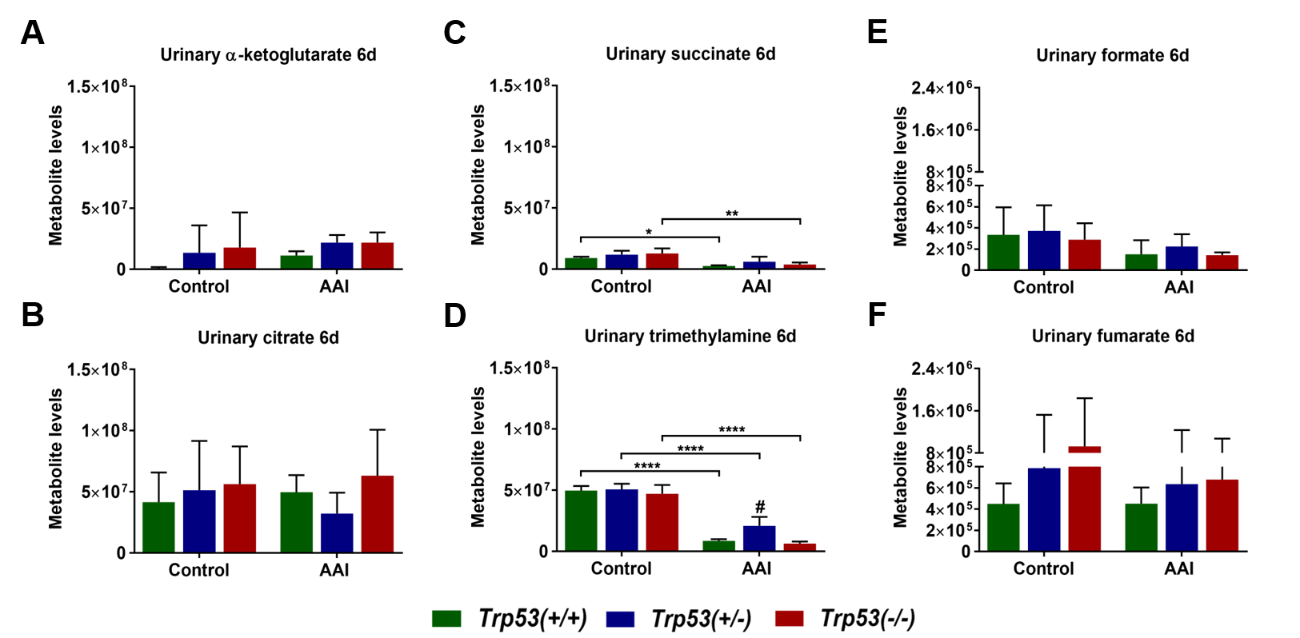

**Fig. S5** DNA adduct formation in liver, kidney and bladder tissues of AAI-treated *Trp53(+/+)*, *Trp53(+/-)* and *Trp53(-/-)* mice (**a**) and *Trp53(+/+)*, *Trp53(+/-)* and *Trp53(-/-)* MEFs exposed to 50 µM AAI for 48 hours (**b**). Representative autographic profiles of DNA adducts are shown. The origins, at the bottom left-hand corners, were cut off before imaging. Spot 1: dA-AAI; Spot 2: dG-AAI; and Spot 3: dA-AAII.

**Table S1** Primers and PCR reaction conditions used to genotype *Trp53(+/+)*, *Trp53(+/-)* and *Trp53(-/-)* mice. More information can be found at [www2.jax.org/protocolsdb/f?p=116:2:0::NO:2:P2_MASTER_PROTOCOL_ID,P2_JRS_CODE:16521,002101](https://www2.jax.org/protocolsdb/f?p=116:2:0::NO:2:P2_MASTER_PROTOCOL_ID,P2_JRS_CODE:16521,002101).


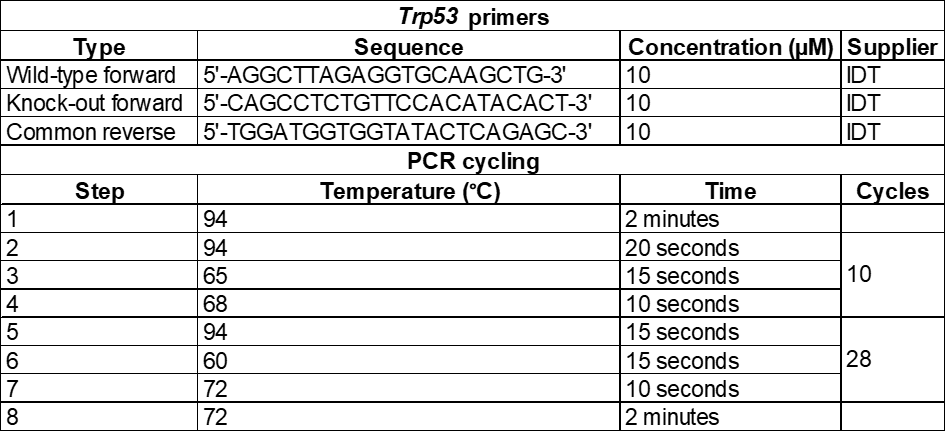


**Table S2** Metabolites detected in the aqueous phase from control and AAI-exposed (6 days) *Trp53(+/+)*, *Trp53(+/-)* and *Trp53(-/-)* kidneys. Results are presented as metabolite levels ± SD (*n* = 5 mice/group). Metabolites in bold are those selected for **Fig. 5**.
